# Statistical Characterization of Received Signal Power for Spectrum-Based GNSS Interference Detection

**DOI:** 10.3390/s26206595

**Published:** 2026-10-17

**Authors:** Krzysztof Bronk, Patryk Koncicki, Adam Lipka, Rafał Niski, Witold Stroynowski, Błażej Wereszko

**Affiliations:** National Institute of Telecommunications, 04-894 Warsaw, Poland; k.bronk@il-pib.pl (K.B.); p.koncicki@il-pib.pl (P.K.); a.lipka@il-pib.pl (A.L.); r.niski@il-pib.pl (R.N.); b.wereszko@il-pib.pl (B.W.)

**Keywords:** GNSS, jamming, spoofing, meaconing, navigation, power variability, interference detection, false alarm probability

## Abstract

This paper presents methods for accurate signal characterization in Global Navigation Satellite System (GNSS) interference detection systems based on spectral analysis of received signals. It specifically characterizes the power variability of signals observed at monitoring stations within such systems. The study is conducted in the context of the currently developed Real-Time GNSS Monitoring System for Poland (RTGMS), whose pilot version was successfully deployed in 2025. The analysis focuses on the impact of power variability of multiple uncorrelated signal components of the received GNSS signals. The results are based on a series of simulations which are validated against measurements collected over several months, encompassing multiple satellite navigation frequency bands. The findings contribute to an improved characterization of signal power variability and highlight its relevance for the design and operation of practical interference detection systems.

## 1. Introduction

GNSS technologies play a significant role in modern world, with substantial impact across numerous domains, including transportation, vehicle fleet monitoring, geodesy, agriculture, cartography, and telecommunications (notably in stations and terminals synchronization). However, in recent years, numerous reports have indicated recurring disruptions in GNSS operation, which can often be correlated with intentional interference events such as spoofing, jamming or meaconing. Such interference incidents are particularly prevalent in regions located near conflict zones (e.g., areas in close proximity to Ukraine and Russia; see, for example, Ref. [1]).

These circumstances highlight the need for the implementation of local GNSS monitoring network systems capable of detecting interference events that may adversely affect the availability and quality of provided services. One such system is RTGMS, which is being deployed nationwide in Poland by the National Institute of Telecommunications (NIT). The development of this system is carried out in collaboration with Poland’s Head Office of Geodesy and Cartography (GUGiK), within a project funded by the European Space Agency (ESA), under NAVISP Element 3 program. There are two key factors that indicate the correct operation of such a monitoring system: (a) reliable detection of destructive interference and/or GNSS signal reception problems within satellite navigation frequency bands and (b) robustness against so-called false alarms. False alarms may arise from misinterpreting natural GNSS signal behavior as interference or from classifying signals that do not significantly affect GNSS performance as severe interference events.

Transmitted signals in radiocommunication systems are subject to numerous propagation effects as they travel from the transmitter to the receiver. This study focuses on interference detection based on properly estimated protection margins derived from an accurate description of received signal power fluctuations. In such interference detection methods, especially when minimizing the probability of false alarms without degrading detection sensitivity, an accurate description of these fluctuations is essential for their performance [2]. To reliably detect intentional interference in low-power systems (e.g., GNSS) and distinguish such events from natural signal fluctuations, the stochastic properties of the received signals must be comprehensively characterized under nominal operating conditions. Therefore, it is crucial to analyze the influence of the propagation environment and the presence of other radio signals at the similar level on the signal at GNSS receivers. This influence manifests itself through signal parameters exhibiting non-deterministic characteristics, which complicates description of GNSS signals and detection of interference after propagation through the radio channel.

The solution proposed in this paper is based on a statistical description of the signal power variability described above, derived from a large number of observed cases and occurrences. Based on this description, an analytical methodology is introduced to minimize the probability of false alarms in GNSS monitoring systems. In particular, this study focuses on defining and characterizing a method for determining optimal parameter values that allow this probability to be minimized and kept at an acceptable level.

This study emphasizes the importance of accurate statistical characterization of signals in GNSS monitoring systems based on spectral analysis. It provides practical values of statistical parameters that can be used to model signal variability at monitoring stations, enabling the design of sensitive systems that remain robust to non-disruptive signals within satellite navigation frequency bands and to inherent power variability in the absence of interference.

This study provides the theoretical background and methodology for evaluating spectral variability in large-scale systems, rather than presenting a full implementation of an interference detection algorithm. Consequently, the focus remains on analyzing received signals rather than comparing disparate systems or algorithms. The primary objective of this work is to highlight the non-uniform power variability that can occur within a single spectral trace.

## 2. Real-Time GNSS Monitoring System for Poland—RTGMS

Although numerous studies address GNSS interference detection and various practical systems monitor the operational status of GNSS [3], significant limitations remain in such systems. A primary issue concerns the scale: globally unified systems, such as GPSJAM [1], often do not prioritize providing real-time information regarding the proper operation of GNSS services, focusing instead on identifying long-term trends of recurring signal disruptions in specific regions. Conversely, combining several small-scale, high-performing local monitoring networks offers a limited ability to aggregate results due to differences in equipment, data types, interference assessment methods, and other operational variations [4]. Recently, particularly in the context of the ongoing conflict between Russia and Ukraine, the deployment of a unified, local GNSS interference detection system operating across a large area (i.e., nationwide) near the conflict zone has become essential.

The authors have been involved in the development of such a system in Poland, namely the mentioned above RTGMS. The most important functions of this system include: (a) detection of intentional and unintentional GNSS signal interference (jamming, spoofing, and meaconing) and/or GNSS signal unavailability in the territory of Poland; (b) generation of alarms indicating instances of navigation system malfunction and (c) real-time visualization of data, alarms, and system status in the RTGMS network via a web-based service accessible to all interested users. The RTGMS is developed based on several key assumptions, two of which address the trade-off between detection range and system reliability: specifically, maintaining a false-alarm probability of 0.1% and targeting a single-station detection range of 25–30 km.

The RTGMS system, developed by the NIT in cooperation with GUGiK, aims to address the need for a highly reliable and accessible platform that informs users about the operational status of GNSS services in Poland. The authors and all parties involved in the project have successfully completed the first stage of the pilot network deployment, which included the integration of more than 20 physical monitoring stations and the launch of a web service for presenting monitoring results.

### 2.1. RTGMS System Assumptions and Characteristics

The RTGMS system consists of stationary, mobile and online components.

The stationary component consists of a network of continuously operating GNSS-band monitoring stations distributed across the entire territory of Poland. These stations utilize the existing infrastructure of the ASG-EUPOS reference station network, which is established and operated by the GUGiK [5]. Currently, the ASG-EUPOS network comprises approximately 150 stations.

The mobile component is essentially a GNSS measurement platform installed on a vehicle and operating in an ad-hoc mode. This platform, referred to as Mobile GNSS Galileo Measurement Platform (MSP2G), was developed in 2022 by the NIT and serves as an advanced mobile laboratory dedicated to field measurements of signal quality and availability. This component enables highly accurate measurements and detailed analysis of complex interference incidents in cases when such analyses would not be possible using solely the stationary one.

Finally, the online component is a dedicated web-based service available without restrictions to all interested users. It provides near-real-time system status information, measurement results, and alarms from all operational GNSS monitoring stations. The first version of this service was deployed at the end of November 2025 and is available at [6].

At this stage, the pilot version of the RTGMS network comprises a limited set of monitoring stations, located particularly at the areas that are most exposed to recurrent interference events within the territory of Poland (eastern border regions, the vicinity of the Kaliningrad Oblast, and the Baltic Sea coast). For this purpose, 24 stations were selected from the available ASG-EUPOS network and integrated into the RTGMS pilot infrastructure. Further system expansion is planned to eventually achieve a full nation-wide coverage.

The RTGMS system is based on a centralized architecture. Data streams from monitoring stations (i.e., the stationary component) are transmitted to a central processing server operating within the ASG-EUPOS network, where core data analysis and interference detection algorithms are executed.

The RTGMS system is designed not only to achieve full nationwide coverage, but also to analyze all major GNSS frequency bands (i.e., E1, E5a, E5b, E5ab, E6 of Galileo; L1, L2, L5 of Global Positioning System (GPS); G1, G2, G3 of GLONASS and B1, B1C/B1A, B2a, B2/B2b, B2, B3/B3A of BeiDou).

One of the most important features of the RTGMS system is its ability to provide reliable and accurate information on interference events within GNSS frequency bands. For this purpose, interference detection is performed at what the authors consider three distinct levels of analysis, referred to as the “high”, “medium” and “low” levels.

High level analysis is based on direct monitoring of decoded PNT (Positioning, Navigation and Timing) data, primarily position information. These data are obtained directly from GNSS receivers. Typical parameters at this level include position error estimates, ground track, Signal-to-Noise Ratio (SNR), the number of visible satellites in a given constellation, Horizontal, Vertical and Time Dilution of Precision (HDOP, VDOP, TDOP) and related indicators. High-level data serve as complementary information in the RTGMS interference detection framework.

Medium level depends on observables (raw data, ephemerides, and pseudo range measurements) used by the receiver to compute the final position solution. This approach allows for a more focused analysis and facilitates the identification of specific frequency bands and GNSS constellations that are particularly susceptible to signal distortions, thereby providing greater accuracy than high-level analysis. Medium-level analysis utilizes RINEX data streams collected from monitoring stations and may also be applied in post-processing mode using dedicated hardware platforms (e.g., the MSP2G measurement vehicle for ad-hoc analysis of selected interference events) or software tools such as RTKLIB [7].

Low level is based on spectral data analysis provided by GNSS receivers installed at RTGMS monitoring stations. All stations generate real-time or quasi-real-time spectrograms that are transmitted to the central RTGMS server, where they are processed using a dedicated interference detection algorithm. The spectral data are compared against a predefined threshold, referred to as the spectrum mask. A signal level exceeding the spectrum mask indicates a potential interference event. In such cases, cumulative signal energy analysis is performed to distinguish short-duration and insignificant disturbances from severe interference events. The low-level analysis constitutes the most critical detection layer within the RTGMS system, as final interference classification decisions are primarily based on this approach.

**Figure 1:**
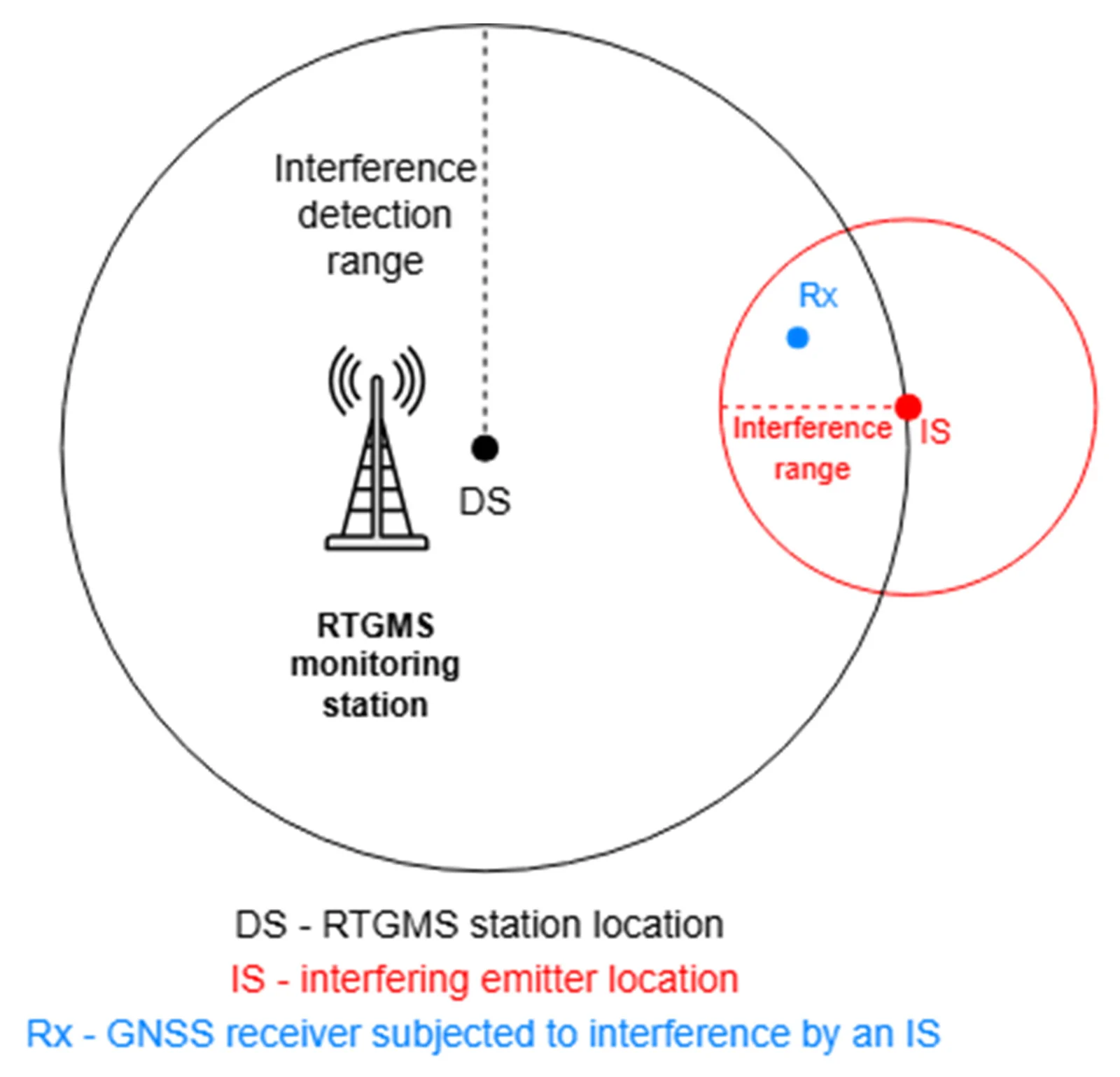
Visualization of the sample interference range and detection range around the RTGMS monitoring station.

High- and medium-level analyses provide information only on interference events that are sufficiently strong to degrade receiver’s performance and that occur in the close vicinity of a monitoring station; therefore, they serve a complementary role within the RTGMS system. On the other hand, the low-level approach, which is based on spectrum analysis, constitutes the core of interference detection in the RTGMS system. This spectrum-stream–based approach enables the detection of interference sources located at considerable distances from the monitoring station. As a result, each monitoring station is characterized by a specific detection range, defining the area within which interference events can be identified. This concept is analogous to that of a cellular network, implying that a relatively small number of monitoring stations is sufficient to achieve nationwide coverage within the RTGMS system. Figure 1 illustrates one possible scenario for detecting interference around a monitoring station using spectral (low level) analysis.

While detection in high- and medium-level analyses is constrained to the physical coverage of the interfering emission, low-level analysis enables a monitoring station’s detection range to significantly exceed the effective operational range of an interfering transmitter under practical conditions. This is because successful GNSS interference—resulting in a receiver’s desynchronization from satellites or the loss of tracking for a significant number of satellites in most cases requires the Interference-to-Signal Power Ratio (I/C) to be substantially higher than the GNSS signal power [3,8,9], and consequently much higher than the minimum detectable I/C level. Interference detection capability of the RTGMS system is further enhanced by the monitoring stations being typically placed on building rooftops at heights that are usually noticeably greater than heights of GNSS receivers, of approximately 10–15 m above ground level (h_DS_ > h_Rx_ in most cases). It provides favorable propagation conditions between the interference source and the monitoring station, thereby increasing the potential for a successful interference detection.

### 2.2. Interference Detection Algorithm in RTGMS System

The core of the low-level analysis in the RTGMS system—i.e., the interference detection algorithm—is based on the analysis of spectral streams collected from selected monitoring base stations. The algorithm is designed to process both individual spectral traces and averaged and/or filtered spectral streams. However, the use of preprocessed spectral data is particularly advantageous, as spectrum averaging and filtering simultaneously reduce the probability of false alarms and significantly improve the sensitivity of the detection algorithm, both of which are critical to overall system performance.

The proposed detection algorithm consists of two fundamental stages, i.e., relative interference power analysis and interference energy analysis. The first stage is performed to identify potential interference events, while the second stage verifies whether the detected events are significant and/or cause actual degradation of GNSS signal reception. Confirmation of interference requires that the interference power exceeds a predefined threshold determined by the spectrum mask and that the accumulated interference energy exceeds an energy threshold within a specified accumulation time window.

Detected interference events are classified using two priority levels:

- Low-priority alarm (yellow alarm);
- High-priority alarm (red alarm).

Exceeding the spectrum mask constitutes a necessary—but not sufficient—condition for generating a high-priority (red) alarm. Such an exceedance is treated as an initial indication that the analyzed system may be subject to a significant interference event, resulting in the generation of a low-priority (yellow) alarm. Final confirmation of the event is performed during the energy analysis stage. If the accumulated interference energy exceeds the predefined threshold, the RTGMS system generates a red alarm and maintains this status at least until the completion of the subsequent accumulation period.

The spectrum mask represents the theoretical expected GNSS signal power level within a selected frequency channel. Under nominal operating conditions (i.e., in the absence of interference), the received GNSS signal should not exceed this level. If such an exceedance occurs, it constitutes an initial indication that an interference incident may be ongoing. However, as noted above, mask violation alone is insufficient to classify the event as a serious interference incident and to generate a high-priority alarm. Only after successful completion of the energy analysis stage can it be classified as severe. When both criteria are satisfied, it is an indication that the incident exhibits both significant power and sufficient duration.

The algorithm operates in two modes:

- Calibration mode, in which the spectrum mask levels are defined;
- Analysis mode, which constitutes the primary operational mode, in which the detection algorithm is applied to data acquired from RTGMS monitoring stations.

**Figure 2:**
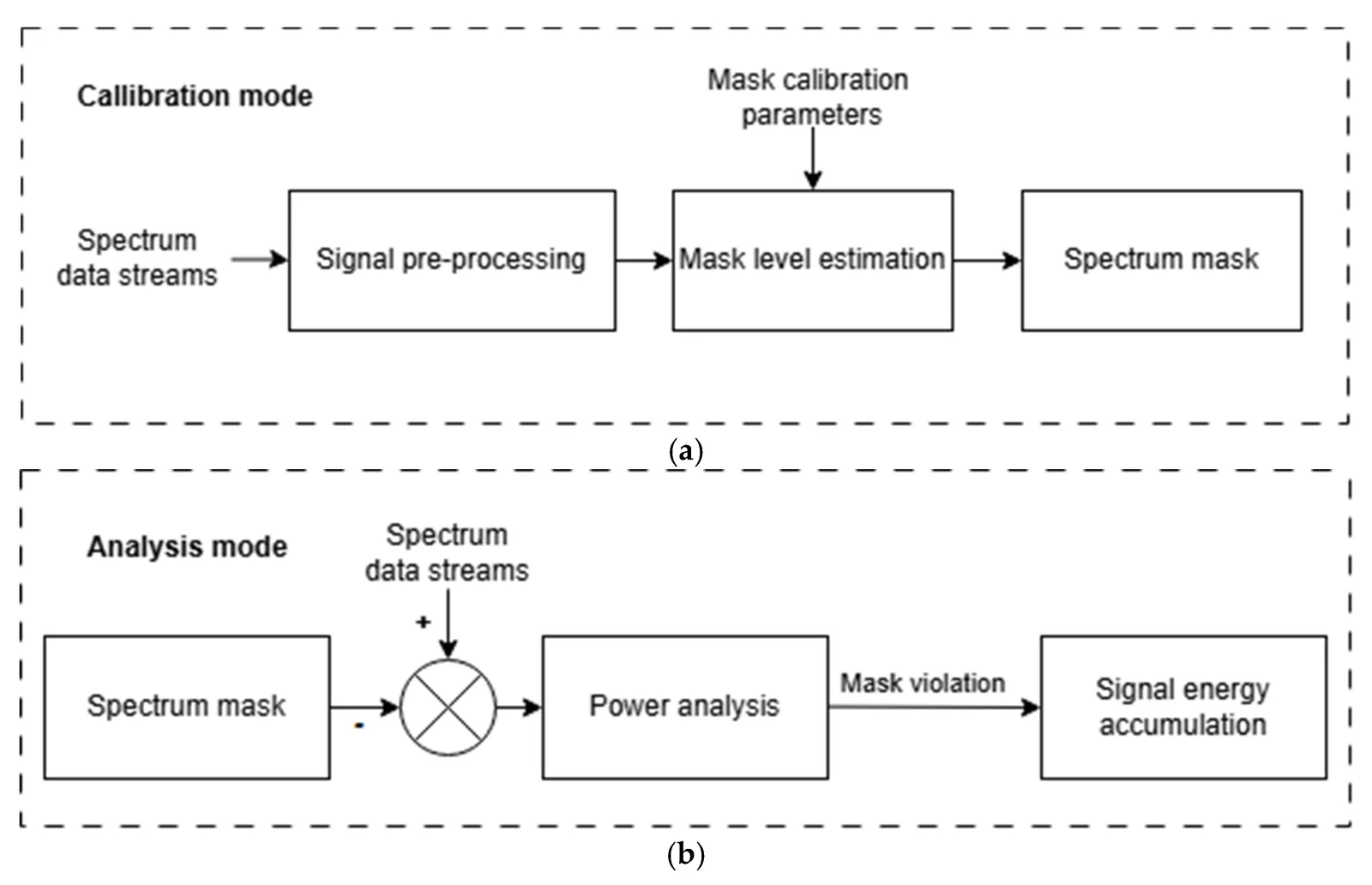
RTGMS interference detection algorithm: (**a**) calibration mode, and (**b**) analysis mode.

A general schematic representation of the algorithm is presented in Figure 2.

The incorporation of the spectrum mask into the algorithm is intended to extract the useful signal component (expressed in terms of the Carrier-to-Noise Ratio, C/N, where C denotes the useful signal power and N denotes the noise power) from the measured spectrum, such that only the component associated with the interference power (denoted as I) remains. This approach is valid only when the interference power is greater than the sum of useful signal power within the Resolution Bandwidth (RBW) and the power of Noise in the analyzed spectrum (I > C + N). Subtracting the spectrum mask from the measured spectral trace therefore results in the extraction of the I/C component, which represents the relative power of the interfering signal.

GNSS signals are known to be received well below the noise floor [10]. To ensure the validity of the method implemented in the power-based RTGMS detection algorithm, the spectrum traces are generated after all signal processing procedures (i.e., filtering, amplification, and averaging), and the levels of the mask and individual traces are compared within individual RBW bands, without distinction between narrowband and wideband signals. Consequently, the algorithm (and, by extension, all assumptions in this study regarding the levels of individual components) is based on observable power levels at the spectrum analyzer rather than their actual power.

Although interference energy analysis plays a crucial role in the algorithm under consideration, its detailed description is not directly relevant to the topic of this study. This is because potential false alarms occur during the power analysis stage of the algorithm, particularly when the mask level is set incorrectly.

## 3. Protection Margin in Power-Based GNSS Interference Detection Algorithms

As previously stated, false alarms in GNSS detection systems are defined as events in which natural GNSS signal level fluctuations and/or radio signals that are insignificant in the context of GNSS reception are incorrectly identified as serious interference incidents. Interference detection algorithms, such as the one implemented in the RTGMS, must ensure continuous system operability while maintaining the false-alarm probability at a minimal level. This can be achieved by increasing the effective level of the aforementioned spectrum mask through the inclusion of a protection margin into the algorithm. However, introducing a protection margin into an interference detection algorithm may lead to adverse effects if not precisely determined. In particular, an excessively high threshold, while improving robustness against false alarms, significantly reduces the probability of detecting actual interference events. Conversely, an overly low threshold, although maximizing detection sensitivity, leads to frequent false alarms that may undermine the credibility of the system’s interference detection capabilities. Accurate determination of this parameter requires not only comprehensive knowledge of all received signal components but also a thorough understanding of the impact of the propagation environment on the spectra analyzed by the algorithm.

To calculate a protection margin value that does not compromise system performance and maintains a low false alarm probability, it is essential to propose methods for the accurate statistical description of received signals.

### 3.1. Statistical Description of Received GNSS Signal Level Variability

The statistical description of received GNSS signals is pivotal in determining the value of the protection margin. This is because the processes to which received signals are subjected to in a real-world propagation environment must be accurately described in order to optimize the value of this parameter. A deterministic description of these processes is not feasible; therefore, their statistical properties must be characterized in accordance with the fundamental principles of probability theory.

Due to the stochastic nature of received signals, their power is most commonly described using probability density functions. The non-deterministic variations in the received GNSS signal power envelope caused by propagation in a real-world environment are, in most cases, assumed to be affected mostly by large-scale fading and modeled using the log-normal distribution [11,12]. The probability density function of a Random Variable (RV) X_LN_ following a log-normal distribution, according to [2], is given by:

(1) $$p_{LN}\left(X_{LN}\right)=\frac{1}{X\sigma_{LN}\sqrt{2\pi }}\exp\left[-\frac{1}{2}{\left(\frac{\ln\left(X_{LN}\right)-\mu_{LN}}{\sigma_{LN}}\right)}^{2}\right],\;\;X>0$$

where µ_LN_ is the mean value of ln(X_LN_), and σ_LN_ is the standard deviation of ln(X_LN_).

One of the most important properties of the log-normal distribution is that for a RV X_LN_ that follows a log-normal distribution, the quantity

(2) $$X_{N}=\ln\left(X_{LN}\right)$$

is a RV described by the normal (Gaussian) distribution, with the probability density function defined in [2] as:

(3) $$p_{N}\left(X_{N}\right)=\frac{1}{\sqrt{2\pi }\sigma_{N}}\exp\left[-\frac{{\left(X_{N}-\mu_{N}\right)}^{2}}{2\sigma_{N}^{2}}\right],\;\;-\infty <X<\infty$$

The Gaussian RV defined in (2) is obtained using the natural logarithm. However, in radiocommunication systems, signal power is most commonly expressed in decibels:

(4) $$Y_{N}={10\cdot \log}_{10}\left(X_{LN}\right)$$

The relationship between two Gaussian RVs defined in (2) and (4) is as follows [13]:

(5) $$Y_{N}=\frac{X_{N}}{\lambda },\;\lambda =\frac{1}{10}\ln\left(10\right)=0.2302585$$

This scaling relationship also applies to the mean and standard deviation of X_N_, denoted by µ and σ, respectively. The corresponding relations enabling the application of (4) are given by:

(6) $$\mu_{Y}=\frac{\mu_{X}}{\lambda }$$

(7) $$\sigma_{Y}=\frac{\sigma_{X}}{\lambda }$$

Since Equation (4) implies that the statistical characteristics of signal power expressed in decibels can be modeled by a normally distributed RV, discrete spectral samples in the subsequent analysis are characterized by their sample mean and standard deviation, defined in [14] as:

(8) $$\mu_{x}=\frac{1}{N_{sampl}}\sum_{n=1}^{N_{sampl}}x[n]$$

(9) $$\sigma_{x}=\sqrt{\frac{1}{N_{sampl}}\sum_{n=1}^{N_{sampl}}{\left(x[n]-\mu_{x}\right)}^{2}}$$

where N_sampl_ is the number of signal power samples and x[n] denotes the sample values.

However, noise and the sum of uncorrelated signal powers may exhibit non-central characteristics. Therefore, to properly describe the signals in further analyses, additional statistical measures are considered. In particular, skewness is used to quantify the asymmetry of the signal power distribution [15]:

(10) $$\gamma_{x}=\frac{m_{3}}{\sqrt{m_{2}^{3}}}$$

where:

(11) $$m_{i}=\frac{1}{N_{sampl}}\sum_{n=1}^{N_{sampl}}{\left(x\left[n\right]-\bar{x}\right)}^{i}$$

and excess kurtosis coefficient $\kappa_{x}$ is used to describe the presence of outliers in the power distribution [15]:

(12) $$\kappa_{x}=\frac{m_{4}}{m_{2}^{2}}-3$$

It should be noted that, in the subsequent analyses, the primary focus is on the variability of the power envelope resulting from large-scale fading. Therefore, the mean signal power is not of primary importance, and only parameters defined in (9), (10) and (12) are considered.

In order to accurately and reliably determine the value of the protection margin in a power-based interference detection algorithm, it is necessary to introduce the concept of confidence intervals for signal power described by specific probability distributions. In the case of a normally distributed RV X_N_, it is possible to determine the probability of this variable assuming any specified value x [14]. A confidence interval is defined by the probability that the variable x lies within a specified range, a ≤ x ≤ b. For a Gaussian RV, this probability can be expressed as:

(13) $$P=\int_{a}^{b}p_{N}\left(X_{N}\right)dX_{N}$$

After appropriate transformations, it is possible to determine this probability for a specified multiple of the standard deviation (i.e., that the Gaussian RV X_N_ lies within the interval −kσ ≤ x ≤ kσ). This probability is expressed using the function erf(k):

(14) $$\mathrm{erf}\left(k\right)=\frac{1}{\sqrt{2\pi }}\int_{-k}^{k}e^{-\frac{z^{2}}{2}}dz$$

where z = (X_N_ − µ_N_)/σ.

It should be noted that (14) accounts for both tails of the distribution. However, in the case of false alarm probability, only the values exceeding the mean are of interest. To obtain the corresponding probability, the limits of integration in (14) must therefore be modified to the interval [k; ∞).

Equations (13) and (14) are defined strictly for Gaussian RVs. Although the received power of the useful transmitted signal (expressed in decibels) can be approximated by a normally distributed RV, the addition of other components to the received signal—such as noise and other radio signals within adjacent frequency bands—can render the overall distribution non-Gaussian. This is one of the reasons why signal averaging is applied in interference detection algorithms.

Averaging of spectral signals allows the Gaussian approximation of the received signal distribution only when the Central Limit Theorem (CLT) is applicable (i.e., when at least 30 spectral traces are averaged [16]). If this condition is not satisfied, confidence intervals are calculated using Student’s t distribution with a number of degrees of freedom equal to the number of averaged spectral traces [17].

Another important consequence of the fact that the received signal consists of multiple components beyond the useful signal is that, in order to precisely determine how the sum of these components shapes the overall received signal distribution, it is necessary to apply appropriate methods for summing independently distributed RVs. Numerous such methods are available in the literature [18,19,20]. In the subsequent analysis presented in this paper, it was assumed that the dominating phenomenon rendering received signals non-deterministic was large-scale fading, and consequently signal components follow log-normal distributions [11,12]. Therefore, this study considers the Log-Normal Methods (namely k-LNM and t-LNM), and Monte Carlo simulations [13].

### 3.2. Protection Margin Parameter in RTGMS System

In general, the GNSS signal received by monitoring stations is considered to be the sum of three independent primary components: C (representing GNSS signal power), I (representing interfering signal power), and N (representing noise power). A similar approach has been adopted in numerous scientific publications [12,21,22,23,24]. As mentioned above, interference is detected by extracting both C and N components so that only the “I” component remains. However, this approach is effective only if the false alarm probability is sufficiently reduced, which is achieved through the introduction of a protection margin.

The protection margin in the RTGMS system is denoted by the parameter “A”. The introduction of this parameter imposes certain limitations on the algorithm’s sensitivity and on the minimum detectable interference power. The achievable algorithm sensitivity (defined as (I/C)_min_) is subject to the following limitation:

(15) $${\left(\frac{I}{C}\right)}_{min}[dB]>A[dB]+N[dBm]$$

Furthermore, with the incorporation of parameter “A” into the algorithm, the absolute minimum detectable interference level relative to the processed power *C* within the same RBW band as the interference becomes:

(16) $$I_{min}[dBm]>C[dBm]+N[dBm]+A[dB]$$

With this formulation, the initial assumption adopted in the algorithm definition, namely that I > C + N, is satisfied, thereby validating the chosen approach in the described interference detection algorithm.

When the statistical parameters of all received signal power components are known, and consequently the parameters of the overall received signal power distribution at the monitoring station can be approximated, it becomes possible to determine the value of the parameter “A” for a specified false alarm probability:

(17) $$A=k\cdot \frac{\sigma }{\sqrt{N_{avg}}}$$

where σ is the estimated standard deviation of received spectral power envelope at the monitoring station, N_avg_ is the number of averaged spectral traces and k is determined using confidence intervals either based on [16]:

- Gaussian distribution in case of at least 30 spectral traces;
- Student’s t distribution with N_avg_ degrees of freedom otherwise.

For both Gaussian- and Student’s t-based confidence intervals, the value of parameter k can be obtained from standard statistical tables [17,25].

When calculating the value of parameter “A” in (17), several assumptions must be made:

- The received signal spectrum must be expressed on a logarithmic scale (decibels);
- The average power levels of all received signal components (C, I, N) are assumed to be of similar magnitude. This is because the algorithm is configured to operate in a manner that ensures a maximum detection range, often extending far beyond the range of the interfering emission;
- During the determination of the spectrum mask level, it is assumed that no malicious transmission is present; therefore, signals C, I, and N are assumed to be mutually uncorrelated.

Equation (17) further emphasizes the importance of signal preprocessing—such as averaging and filtering—prior to mask level determination. This is because all procedures performed at this stage reduce the value of parameter “A”, which, as indicated by (15) and (16), increases the detection sensitivity of the algorithm without compromising the false alarm probability.

**Figure 3:**
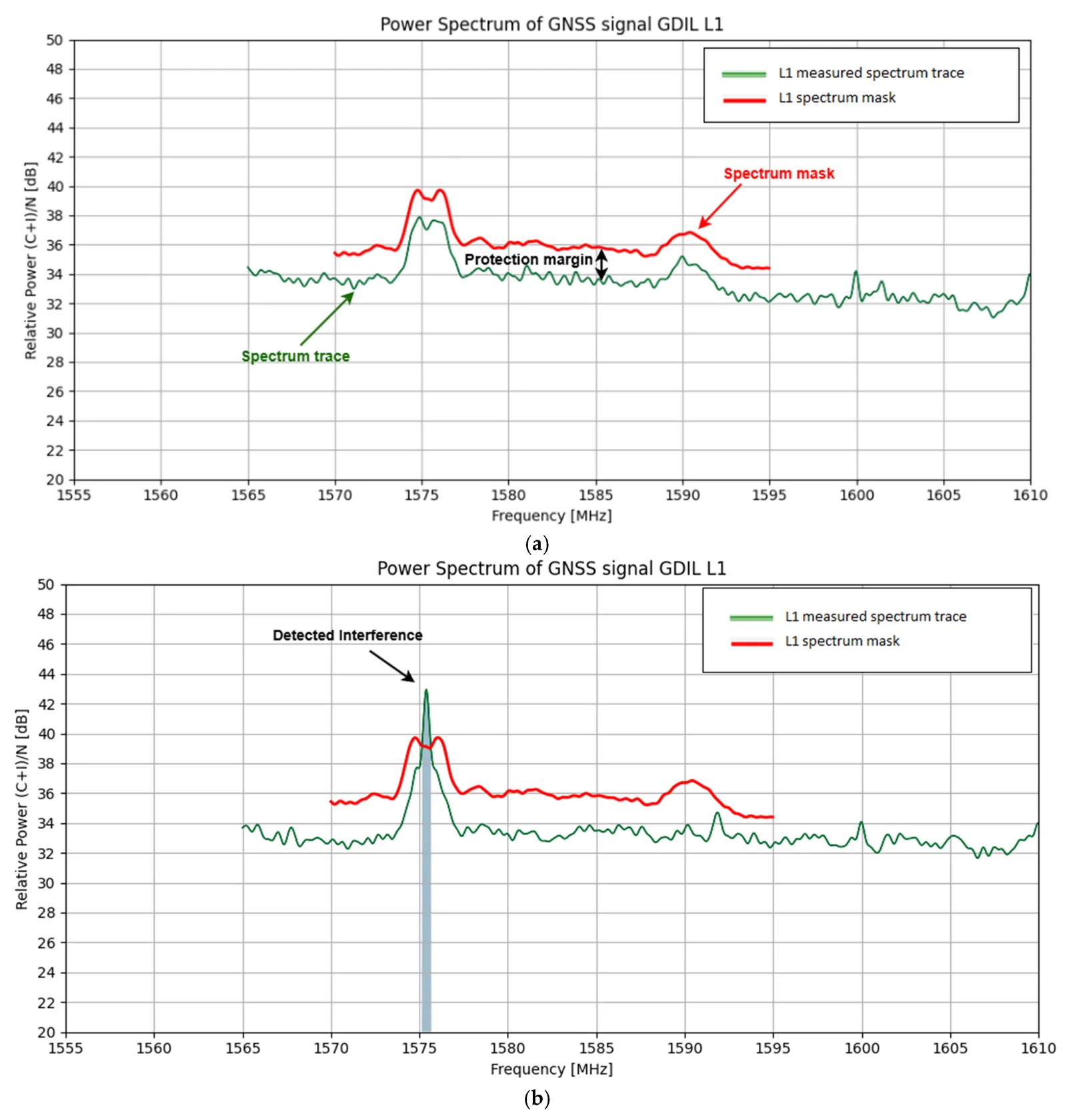
Visualization of interference detection (1st stage of the RTGMS detection algorithm); (**a**) nominal conditions, (**b**) detected interference.

After determining the reference signal level under non-interference conditions during the calibration stage of the algorithm and applying an appropriately calculated spectral mask, interference in the GNSS bands is detected and visualized in the RTGMS system, as demonstrated in Figure 3.

The spectral traces are expressed in terms of relative power levels ((C + I)/N), which relate the absolute values ((C+I)_RBW_) to a specific signal bandwidth (i.e., RBW) and the noise spectral density, as given by:

(18) $$\left(C+I)/N\left[dB\right]={\left(C+I\right)}_{RBW}\left[dBm\right]-\left(-174\left[\frac{dBm}{Hz}\right]\right)-10\cdot {log}_{10}(RBW\left[Hz\right]\right)$$

## 4. Simulation Findings

The reliability of the estimated standard deviation of the received GNSS signal power is crucial for determining the optimal value of the aforementioned margin parameter “A” and, consequently, minimizing the false alarm probability without compromising detection sensitivity. With this in mind, and under the assumption that the resulting standard deviation of the received signal power envelope primarily depends on the power variability of all composite components (C, I and N), it is necessary to determine how the summation of their respective power distributions influences the resulting statistical parameters.

To maximize the reliability of the obtained results, extensive simulations should be conducted and multiple representative scenarios should be considered.

The summation of probability distributions representing the expected composite signal powers after propagation in a real environment was performed in order to:

- Visualize the resulting parameters of the power envelope obtained from the sum of composite signal power distributions within a singular RBW band;
- Compare the summation methods and evaluate their accuracy;
- Visualize the resulting parameters of the power envelope obtained from the sum of composite signal power distributions based on the spectrum analysis of stochastic signals.

### 4.1. Simulation Methodology

All simulations were conducted based on the assumption that, in order to detect interference, the spectrum mask must be exceeded in at least one RBW of the analyzed band. Considering this, along with the fact that the majority of effects on the GNSS signal power envelope stem from large-scale fading [11,12], this study focused on determining the combined effect of the C, I and N components on the stochastic aspects of the resultant signal within a single RBW of the analyzed spectrum. Because the research presented in this study focuses on determining whether power variability across all RBW bands can be classified as statistically insignificant or suspicious, only the power variability of the resultant signal was analyzed. Consequently, the modulation schemes of the received signals were deliberately omitted, and the resultant signal power was characterized by the summation of RVs utilizing three distinct approaches (i.e., Monte Carlo simulations of summed power distributions, the sum of RVs based on LNM methods, and Fast Fourier Transform (FFT) calculation of stochastic signals results).

The simulations were also based on the assumption that a log-normally distributed RV representing signal power distribution becomes Gaussian when the power is expressed in decibels (Equation (4)). To calculate the parameters of the resulting distribution, it was assumed that the power of both C and I components is log-normally distributed [11], and that their distributions can be characterized by their mean power and standard deviation, both expressed in decibels.

While the mean power levels of all three components (C, I and N) varied throughout the simulations, the standard deviation values for the I and C components were selected to replicate signal behavior under realistic propagation conditions. Under conditions similar to those observed in the RTGMS system, the literature suggests that the standard deviation corresponding to the interference component power, σ_I_, ranges from 4 to 6 dB [26]. Therefore, to cover the most representative scenarios, a value of σ_I_ = 5 dB was adopted in the following simulations.

Studies indicate that in case of the standard deviation of the GNSS component power, σ_C_, some variability depends on the frequency band and specific environmental conditions. However, since this variability is relatively low (on the order of approximately 1 dB), an average standard deviation value of σ_C_ = 3 dB was assumed [27].

It should be emphasized that the primary purpose of these simulations was to evaluate how mean value discrepancies affect the summation of components with differing power variabilities; thus, the parameter values representing the RTGMS operational conditions serve a complementary, illustrative role.

Under these conditions, in Monte Carlo method, instantaneous power samples of both the I and C components, denoted by P_I_ and P_C_, were randomly generated in the decibel domain. The generated samples were drawn from the following distributions:

(19) $$X_{P_{I}}∼N\left(\mu_{I},\sigma_{I}\right)$$

(20) $$X_{P_{C}}∼N\left(\mu_{C},\sigma_{C}\right)$$

The power samples of the component representing the noise power distribution, P_N_ were calculated as the square of randomly generated Additive White Gaussian Noise (AWGN) amplitude samples of unit standard deviation scaled by the mean noise power:

(21) $$P_{N}(n)\left[mW\right]={\left(A_{0}(n)\cdot \sqrt{10^{\frac{\mu_{N}\left[dBm\right]}{10}}}\right)}^{2}$$

where A_0_(n) are randomly generated samples from X_N_ ∼ N(0, 1) and µ_N_ is the mean noise power expressed in decibels.

In order to properly simulate the summation of power components under realistic conditions, all generated samples were converted to Watts prior to summation.

All power components were then summed, and the obtained value was converted back to the decibel scale in order to determine the statistical parameters (9), (10) and (12) of the resulting power distribution:

(22) $$P_{sum}(n)\left[dBm\right]=10\cdot {log}_{10}\left(P_{C}(n)\left[mW\right]+P_{I}(n)\left[mW\right]+P_{N}(n)\left[mW\right]\right)$$

The Monte Carlo Simulations were conducted over 30 independent runs, with each run generating power vectors consisting of 2 million random samples.

Although two of the composite components (C and I) of the received GNSS signal are commonly described by log-normal probability distributions, and despite the existence of multiple analytical methods for determining the sum of log-normally distributed RVs, a selected group of these methods (namely the LNM-based methods: k-LNM and t-LNM) was compared with Monte Carlo simulations. The objective was to verify whether the assumption that the resulting power envelope distribution is also log-normal can be justified.

Both k-LNM and t-LNM methods were implemented based on the theoretical framework presented in [13,20]. In the case of Monte Carlo simulations, the distributions were implemented consistently with the simulated time-domain power distributions; specifically, normally distributed random samples (representing the power of C and I signals expressed in decibels) were summed together and combined with randomly generated AWGN power samples (also expressed in the logarithmic domain).

Spectral analyses were based on the FFT of stochastic signals representing the variability over time within a singular RBW band of the received signal of all received composite components. To simulate a single power spectrum trace of the summed components, noise-like time-domain signals with predefined statistical power parameters were generated, converted to a linear scale, and processed using a scaled FFT at a sampling frequency of 50 MHz, with the transform length varied across simulations to produce the linear amplitude spectrum X(k). The linear power spectrum X_p_(k) was obtained by squaring the linear amplitude spectrum:

(23) $$X_{P}\left(k\right)\left[mW\right]=X{\left(k\right)}^{2}$$

Spectra processed in RTGMS system are averaged to reduce signal variability and minimize the required protection margin level. Two averaging methods (i.e., linear and logarithmic) are commonly implemented in practical spectrum analyzers, and both approaches were simulated in this study.

In the simulations, spectral averaging was performed by generating N_avg_ independent time-domain signal frames, computing the spectrum for each frame, and averaging the results in either linear or logarithmic domain.

For linear averaging, the averaged spectrum was obtained by averaging the generated spectral traces in the linear scale and subsequently expressing the result in decibels:

(24) $$\bar{X_{P}}\left(k\right)\left[dBm\right]=10\cdot {log}_{10}\left(\frac{\sum_{i=1}^{N_{avg}}X_{Pi}\left(k\right)\left[mW\right]}{N_{avg}}\right)$$

Logarithmic averaging was performed by first converting individual spectral trace samples to decibels and then calculating their arithmetic mean:

(25) $$\bar{X_{P}}\left(k\right)\left[dBm\right]=\frac{\sum_{i=1}^{N_{avg}}10\cdot {log}_{10}\left(X_{Pi}\left(k\right)\left[mW\right]\right)}{N_{avg}}$$

Regardless of the averaging method applied, the statistical parameters defined in (9), (10) and (12) were calculated based on the resulting averaged spectrum given by (24) or (25).

### 4.2. Simulation Results

As suggested in [28], in the case of summing log-normal distributions with equal standard deviations, the resulting distribution may be approximated as log-normal. However, simulations conducted in this study to compare distribution summation methods, in addition to identifying the most reliable approach, also highlighted the importance of evaluating not only the standard deviation of the resulting distribution but also other shape parameters, particularly when the summed component distributions are characterized by different standard deviations. The reliability of the analyzed methods was assessed by summing two log-normally distributed RVs with identical mean values and with an increasing difference between their standard deviations, which were initially both equal to 3 dB. Errors in the mean values and standard deviations obtained using the LNM methods, relative to the results of Monte Carlo simulations, together with the corresponding skewness and kurtosis values derived from the same simulations, are presented in Table 1 and Table 2.

The t-LNM method enabled fully accurate estimation of the mean and standard deviation of the resultant power distribution. In the case of the k-LNM method, certain inconsistencies between the parameters derived from Monte Carlo simulations and those obtained using this method were observed. These inconsistencies increased proportionally with the growing difference between the standard deviations of the signal components.

However, despite the very accurate predictions of the standard deviation obtained using the t-LNM method, increasing differences between the input standard deviations resulted in skewness and kurtosis values indicating that the fundamental assumption underlying the LNM methods—namely that the sum of log-normally distributed RVs is also log-normally distributed [20,29]—is not strictly accurate in the given case.

**Figure 4:**
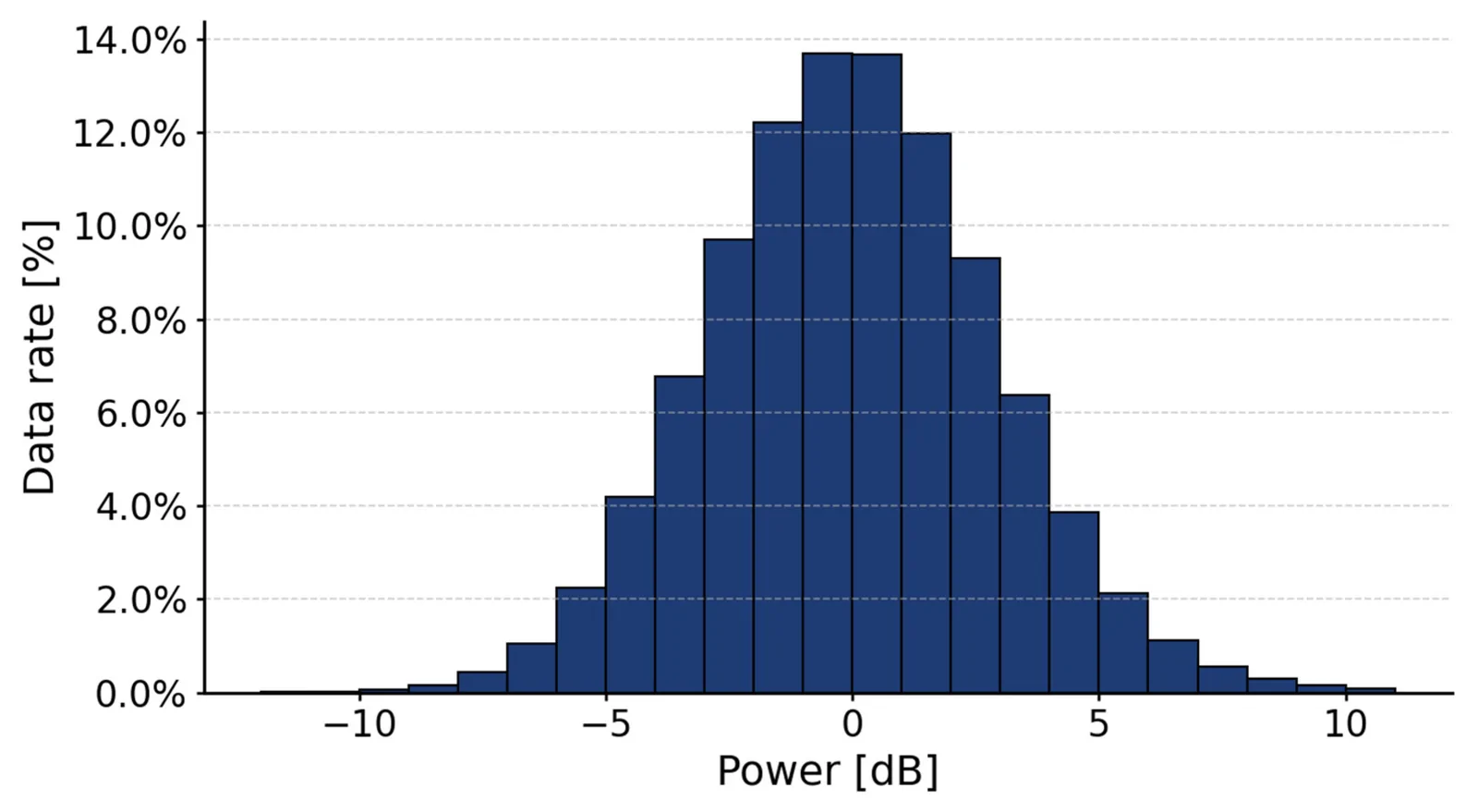
Histogram of simulated power sum for equal mean values of all components.

Monte Carlo simulations were conducted so that three uncorrelated random distributions were generated and summed according to the methodology defined in Equations (19)–(22). The histogram of the simulated power envelope, normalized to a mean value of 0 dB and assuming equal mean power levels for all three signal components, is presented in Figure 4.

The presented histogram is characterized by negligible skewness (10) and kurtosis (12), which indicates that the resultant power distribution can be approximated as Gaussian, with a standard deviation (9) of approximately 3 dB.

**Figure 5:**
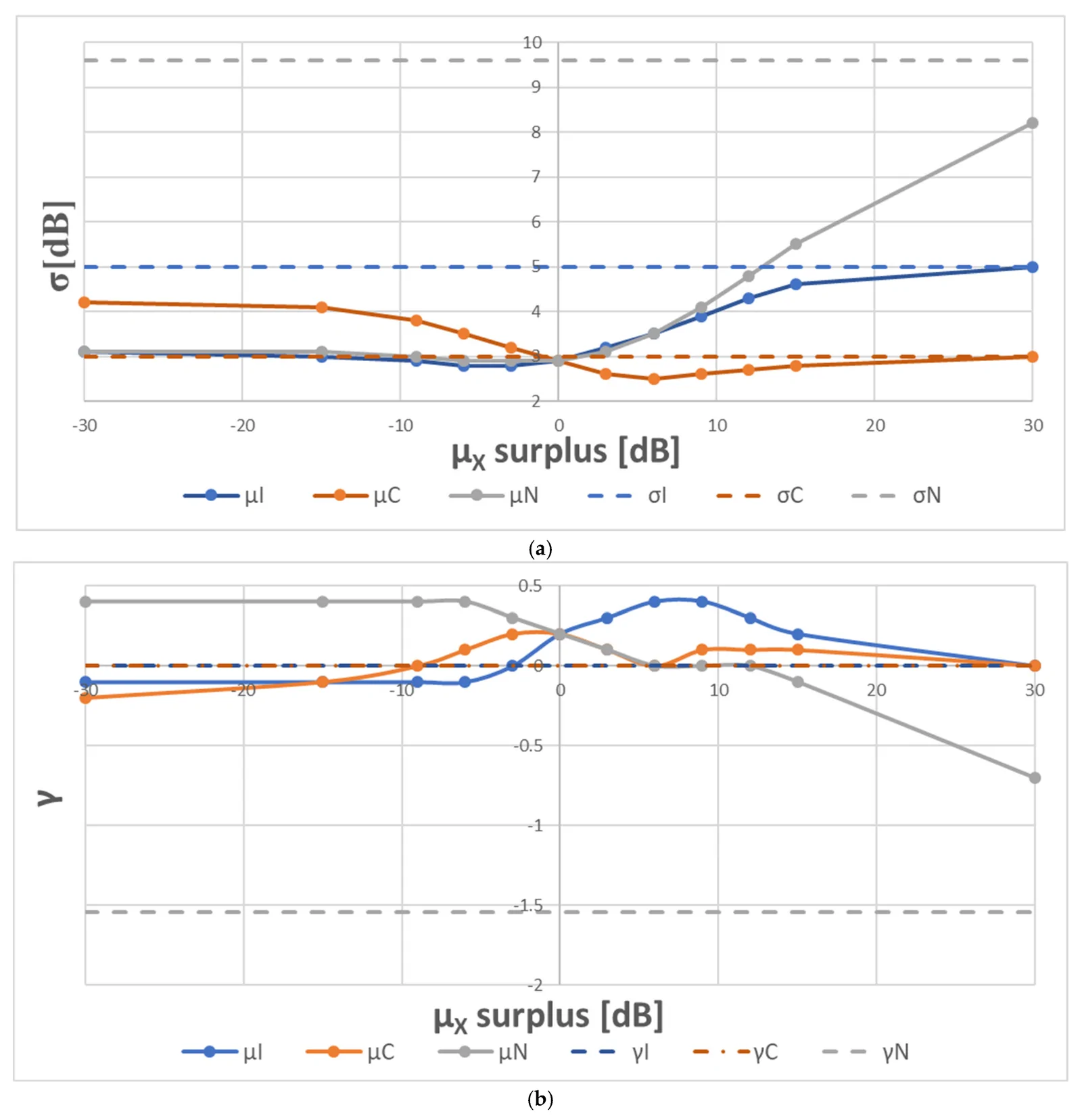

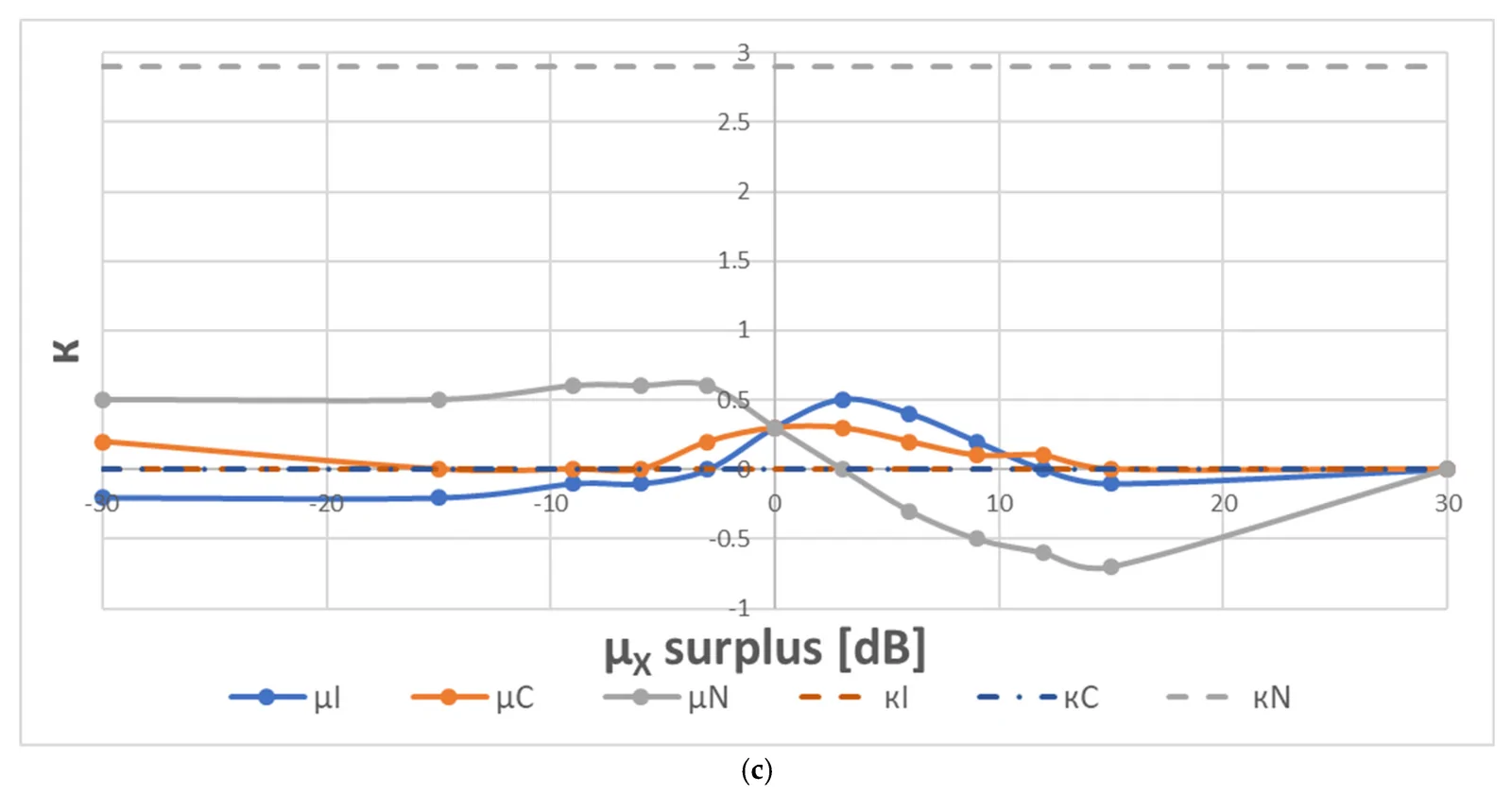
Influence of mean power variability of all components on (**a**) standard deviation, (**b**) skewness and (**c**) kurtosis of the resultant distribution.

Another important characteristic of the simulated distribution sum that was examined was the effect of differences in the mean power of the signal components. In particular, the analysis investigated how significantly the changes in the power of one component affect the shape parameters of the resulting power distribution. Three scenarios were considered, each corresponding to variations in the mean power of one component while the remaining two components were kept at the same level. These simulations enabled observation of both minor and significant changes in the power level of each component, representing specific sub-bands within the spectrum (in which component mean power levels may vary) and/or natural variations in component mean power that occur under normal conditions and should not cause the interference detection algorithm to behave differently. Results of these simulations are illustrated in Figure 5a–c. In all these figures, the parameters of the final distribution (resulting from the power sum of all three components) are assigned to the component with varying mean power in the sum (µ_I_, µ_C_, µ_N_), and the parameters of the individual power distributions of the composite signals are denoted by the corresponding indexed symbols: (a) σ_I_, σ_C_, σ_N_ (b) γ_I_, γ_C_, γ_N_ and (c) κ_I_, κ_C_, κ_N_. The difference between the varying component’s mean power and the constant mean power of the remaining input signals is denoted as μ_x_ surplus.

These analyses indicate that minor fluctuations (differences of up to 5 dB) in the power level of a single component, regardless of which signal is stronger or weaker than the others, do not significantly affect the parameters of the resultant distribution (i.e., the change in the resultant standard deviation, relative to the sum of components with equal means, is less than 0.5 dB). Furthermore, it can be observed that the summation of components reduces the influence of extreme values, resulting in low kurtosis, even in noise-dominated distributions. Another important observation is that as the mean power of one component increasingly exceeds that of the others, the resultant distribution gradually approaches the statistical characteristics of the component with the highest mean power.

Based on these simulations, assuming a standard deviation of the resultant power distribution of 3 dB, the value of parameter k derived from Student’s t tables for a 0.1% false alarm probability and 25 averaged spectral traces results in parameter A, calculated according to (17) being approximately *A* ≈ 2.1 dB.

Simulations based on the FFT algorithm were conducted under the assumption that only large-scale fading significantly affects the resultant power distribution. Therefore, it was assumed that the stochastic characteristics resulting from this phenomenon are sufficient for studying the variability of a singular RBW band in the received signal power under interference-free conditions. For this reason, only non-deterministic signals with predefined statistical distributions were simulated.

Based on the FFT simulations of stochastic signals, it was observed that the parameters of the individual signal components do not affect the resulting spectrum shape, regardless of the number of averaged spectral traces. This leads to the conclusion that, in the case of signals without a deterministic component, even when summing uncorrelated signals with different power distributions, only the mean power of the resulting spectrum is affected. Consequently, this observation leads to a conclusion that can seem contrary to that obtained in the Monte Carlo simulations, namely that when determining the power distribution of the resultant signal, the influence of the power distribution parameters of its individual components is negligible. However, even if received signals are close to the noise floor, the spectrum shape of individual components differs from noise. Therefore, in case of FFT simulations, it may be necessary to examine the influence of modulation schemes on the resultant distributions of the signal.

Another aspect of the FFT analysis that does not influence the shape parameters of the resultant distribution of stochastic signals based on conducted simulations is the spectral resolution; that is, regardless of the number of FFT samples, the power distribution remains unchanged.

**Figure 6:**
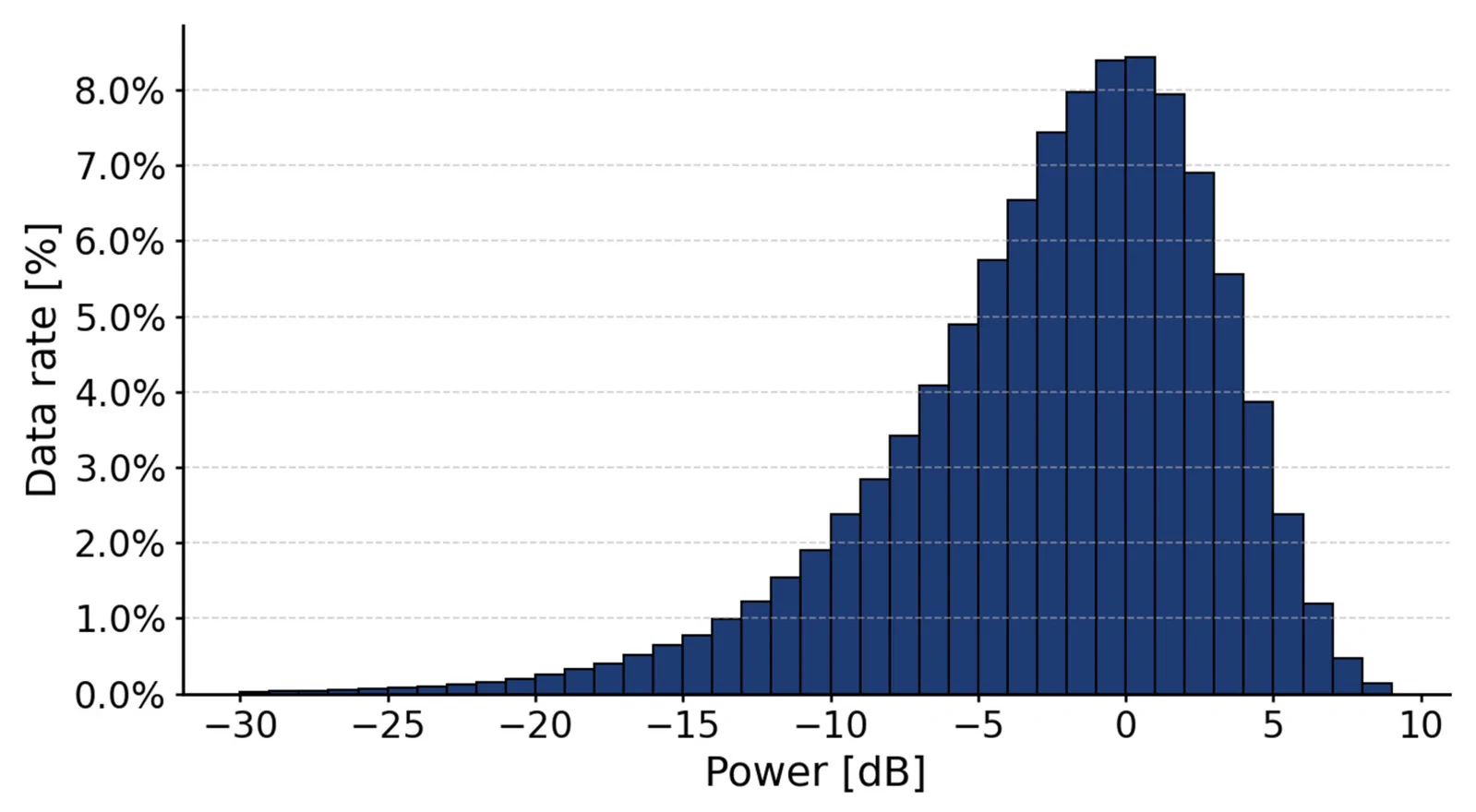
Representative power spectrum histogram of a stochastic signal.

The primary factor that significantly affects the shape of the spectral power distribution when it solely consists of stochastic components is the number of averaged spectral traces. In the absence of averaging (i.e., for a single spectral trace), and for a sufficiently large number of signal samples, the FFT calculation results in a distribution characterized by a standard deviation of approximately *σ* ≈ 5.57 dB and a significant negative skewness. A representative histogram of the spectral power distribution of a resultant signal received from power summation of stochastic components is presented in Figure 6.

The influence of the number of averaged spectral traces on the resultant spectral power distribution can be characterized separately for logarithmic and linear averaging. If the spectra are averaged in logarithmic domain, the relationship can be approximated by the empirical formula:

(26) $$\sigma \left[dB\right]\approx \frac{5.57}{\sqrt{N_{avg}}},\;\;N\geq 1$$

For linear averaging, standard deviation is lower and Equation (26) becomes:

(27) $$\sigma \left[dB\right]\approx \frac{4.35}{\sqrt{N_{avg}}},\;\;N>1$$

**Figure 7:**
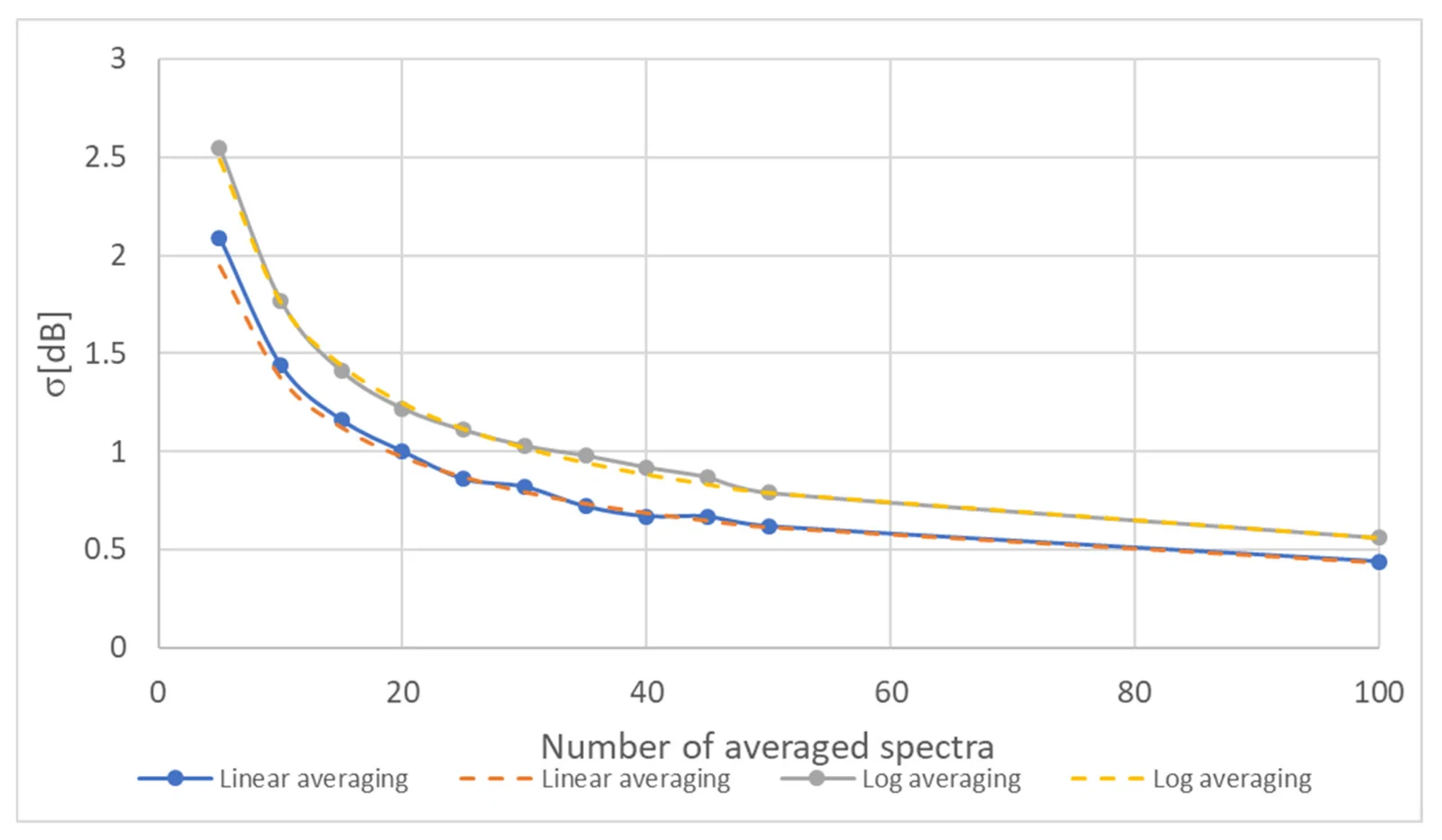
Comparison of the standard deviation regression across the number of averaged spectra for the Keysight formulae (dashed lines) and the simulations (solid lines).

Both Equations (26) and (27) are also suggested in a Keysight application note for noise-like signals [30]. Figure 7 presents a comparison between the curves obtained from simulations and those provided in the Keysight application note.

As predicted by the CLT, averaging also affects both the skewness and kurtosis of the spectral power distribution. As the number of averages increases, both parameters gradually approach zero. This observation also justifies determining the value of parameter k in (17) using Gaussian distribution tables when a sufficiently large number of spectral traces is averaged. The dependence of skewness and kurtosis on the number of averaged spectral traces is presented in Table 3.

Based on the FFT analysis, assuming the received GNSS signal to be noise-like and considering 25 averaged spectral traces, the value of margin parameter A is calculated as approximately A ≈ 3.9 dB.

## 5. GNSS Spectrum Power Variability Measurements

To validate the statistical assumptions derived from simulations and theoretical analysis, real GNSS spectrum measurements were conducted using monitoring stations of the RTGMS network. The purpose of these measurements was to characterize the variability of received GNSS signal power under real operating conditions and to verify the parameters used in the interference detection model, particularly the standard deviation of signal power and the resulting protection margin parameter.

The advanced GNSS receivers installed at the ASG-EUPOS stations used in the RTGMS network are equipped with spectrum analyzer modules that enable observation of the variability of received signal power spectra. The stations operate receivers from three different manufacturers—Trimble, Leica, and Septentrio.

### 5.1. Measurement Procedure

**Figure 8:**
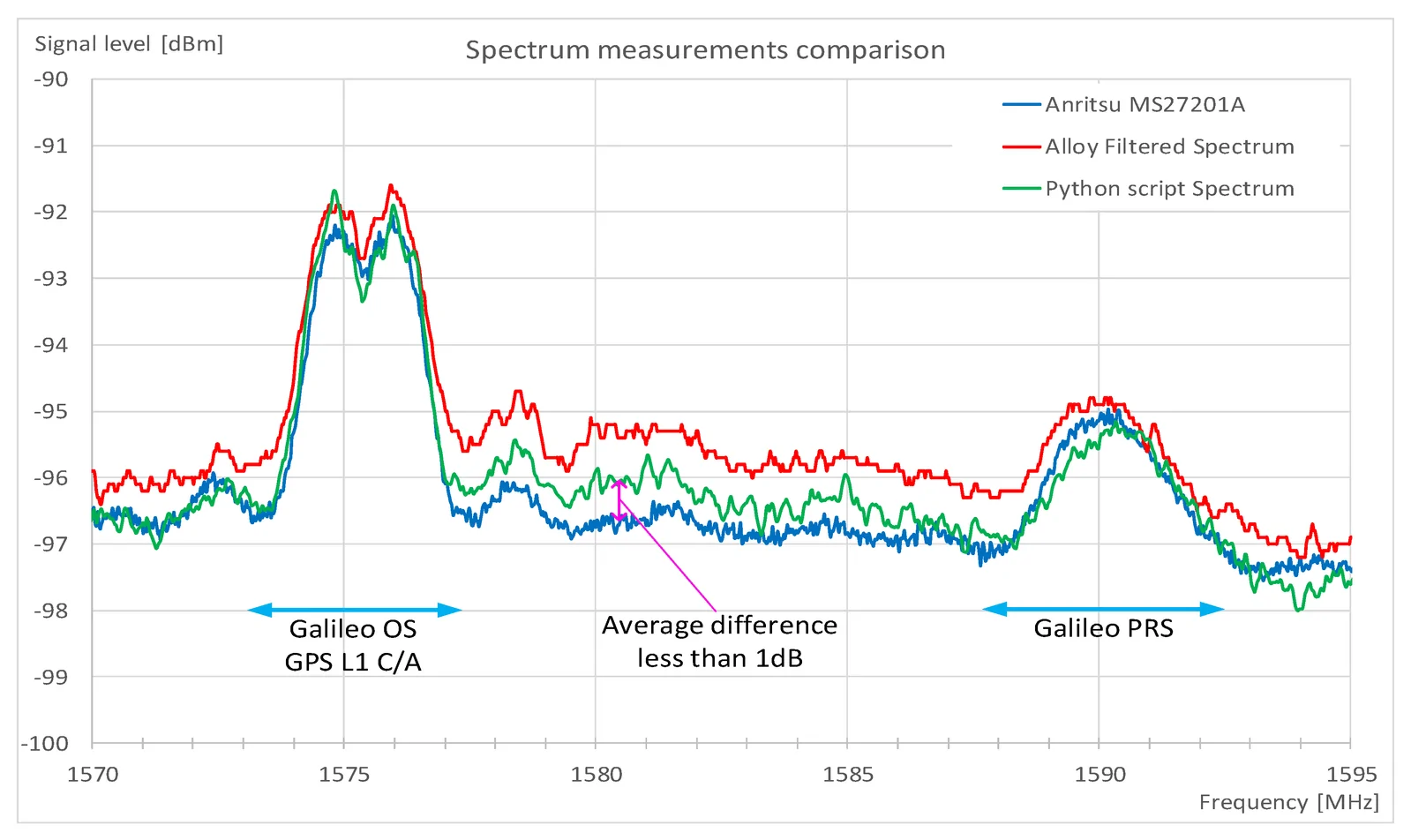
Results of spectra comparison acquired from Anritsu MS27201A spectrum analyzer and Trimble Alloy spectrum analyzer module.

To maximize the accuracy of the measurement results, all receivers available at the RTGMS stations were tested. Due to the accessibility and high reliability of their spectral data, the data used in this study were acquired from Trimble Alloy receivers using a custom Python 3.11 script that interfaces with their integrated spectrum analyzer module. This script collected raw data samples from the receivers and performed linear-scale averaging in the time domain. Prior to further analysis, the Python-derived spectral traces were validated against measurements from an Anritsu MS27201A spectrum analyzer, demonstrating an average discrepancy of less than 1 dB. A representative test example of the spectrum analyzer module in the Trimble Alloy GNSS receiver is illustrated in Figure 8.

**Figure 9:**
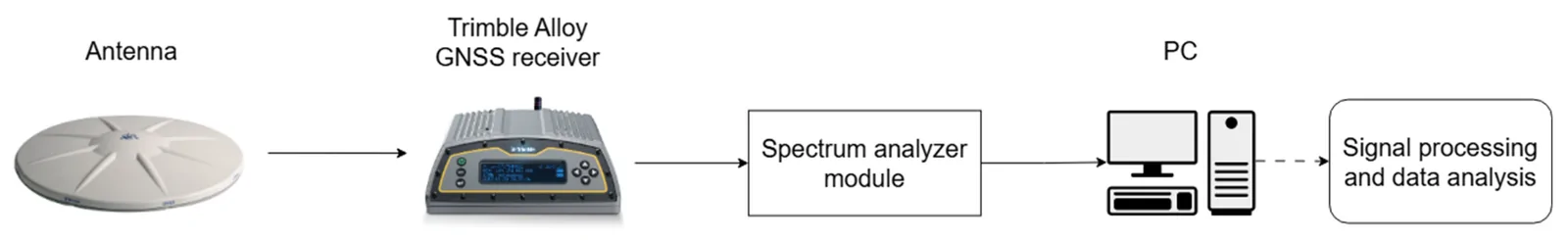
Spectrum measurement block diagram.

All spectral streams were measured using receivers located at reference monitoring stations within the RTGMS system. A general measurement scheme is presented in Figure 9 [31,32].

Statistically analyzed measurements of the spectral streams were collected from the spectrum analyzer modules of receivers installed at five locations in northern Poland: Bartoszyce, Gdańsk, Braniewo, Suwałki, and Wicko, and for multiple frequency bands (i.e., E1/L1, L2, L5 and E6). However, extensive quantitative analysis was conducted primarily for the E1/L1 band. The analyzed dataset included measurements conducted during February, March, April and September of 2025.

Data obtained from the spectrum analyzers were stored in *.log* files containing spectral samples together with metadata describing the collected streams (such as station location, frequency band, number of averaged spectral traces, etc.). The recorded data were subsequently processed using Python-based software, in which the spectral power data were analyzed from a statistical perspective for selected channel bandwidths using the methodology described previously (i.e., standard deviation, skewness, and kurtosis were calculated). The same software environment also enabled visualization of the received spectra and generation of histograms of spectral data for selected time intervals.

Each spectral data stream was acquired from spectrum analyzer modules integrated into Trimble Alloy receivers, yielding 2048-point FFTs across a 50 MHz bandwidth at update rates of up to 5 Hz, corresponding to a RBW of 24.414 kHz. Actual data collection rates varied depending on network infrastructure limitations and link capacities, as most receiver data were accessed remotely via a request/pull mode. Additionally, time intervals were selected to ensure non-overlapping spectra. Prior to spectral analysis, all data streams were averaged on a linear scale over a 30-s period. Therefore, in order to extract statistical information about the power variability of a single spectral trace—required for the estimation of the previously defined margin parameter “A”—the histograms representing this variability (ΔP) were rescaled by multiplying them by a factor of √N_avg_:

(28) $$∆P\left[dBm\right]=\left(P\left[dBm\right]-\bar{P}\left[dBm\right]\right)\cdot \sqrt{N_{avg}}$$

where P are the instantaneous spectral traces, $\bar{P}$ are the averaged traces, and N_avg_ denotes the number of averages [16].

The vast majority of analyzed spectra were selected from periods during which no significant interference events were observed. This selection enabled observation of the natural behavior of the signals and ensured that the assumptions required for accurate estimation of margin parameter “A” were satisfied. However, certain spectra were measured during particular interference events, in order to observe how selected types of interference affect the received signals.

The collected data were analyzed primarily to examine the impact of different spectral regions within the GNSS bands on the variability of the received GNSS signal power.

### 5.2. Measurement Results

The analysis of the collected data in the relevant frequency bands (i.e., E1/L1, L2, L5 and E6) indicated only minor differences in signal power variability between these bands under interference-free conditions. However, the E6 band was observed to be more frequently affected by certain types of interference, most likely because this band is shared with amateur radio services [33]. Although, according to ITU-R Radio Regulations, satellite radionavigation is a primary service in the E6 band, amateur transmissions are often observed at power levels that may disrupt GNSS operation. This observation however is not particularly relevant to the topic of the study, as the main goal of presented research is to determine an accurate power variability description without the presence of harmful interference.

Although the differences in signal variability between the analyzed GNSS bands were negligible, examination of sub-bands within these frequency ranges revealed statistical differences between spectral regions in which different signal components dominate. This difference was presented through comparison of spectrum portions corresponding to the GNSS Open Service (OS), including Galileo, GPS and Beidou (approximately 4 MHz around the E1 band center frequency) and the lowest power portion of the spectrum, interpreted as a noise-dominated sub-band in the range of 1600–1604 MHz.

**Figure 10:**
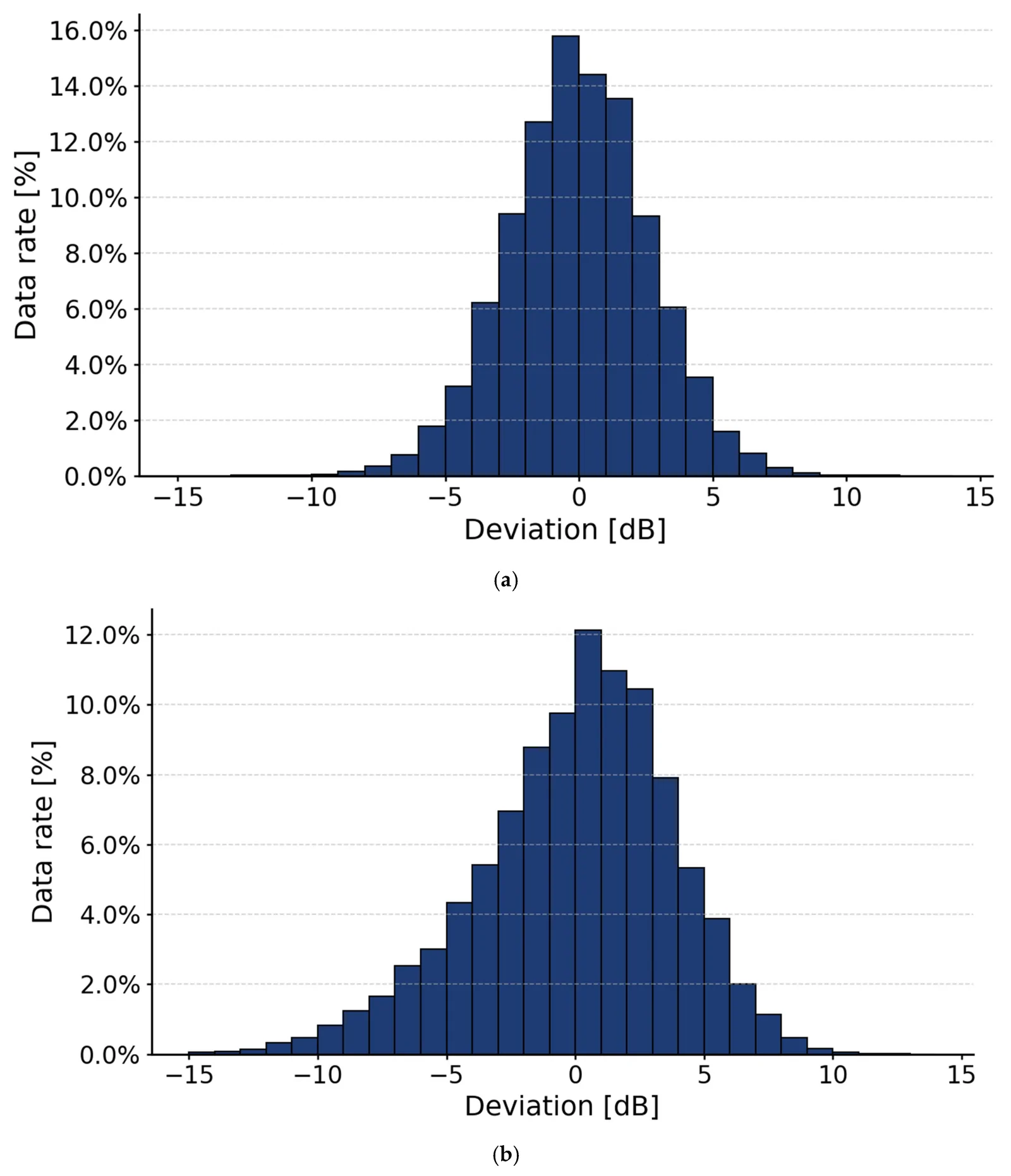
Power histograms for 4 MHz sub-bands of (**a**) GALILEO OS, and (**b**) noise in E1 band. Bartoszyce, 5 April 2025.

The power distribution of the 4 MHz GNSS OS sub-band was observed to follow a log-normal distribution, characterized by negligible skewness and kurtosis. In contrast, the 4 MHz noise-dominated sub-band exhibited negative skewness and non-zero kurtosis. Furthermore, the noise-dominated sub-band was characterized by a larger standard deviation compared to the signal-dominated band. It is likely that the differences in skewness and kurtosis are, in reality, more pronounced, as the spectral traces obtained from the spectrum analyzer modules represent spectra averaged over a 30-s period. According to the CLT, such averaging leads to distributions exhibiting increasingly Gaussian characteristics as the number of averaged spectra increases. Although in the case of standard deviation the effect of averaging can be compensated for by scaling as in (28), its impact on skewness and kurtosis is irreversible. Nevertheless, since observable differences remain, they are illustrated by power histograms presented in Figure 10. Both of them were scaled as in (28), and generated using power measurements collected in Bartoszyce on 5 April 2025.

**Figure 11:**
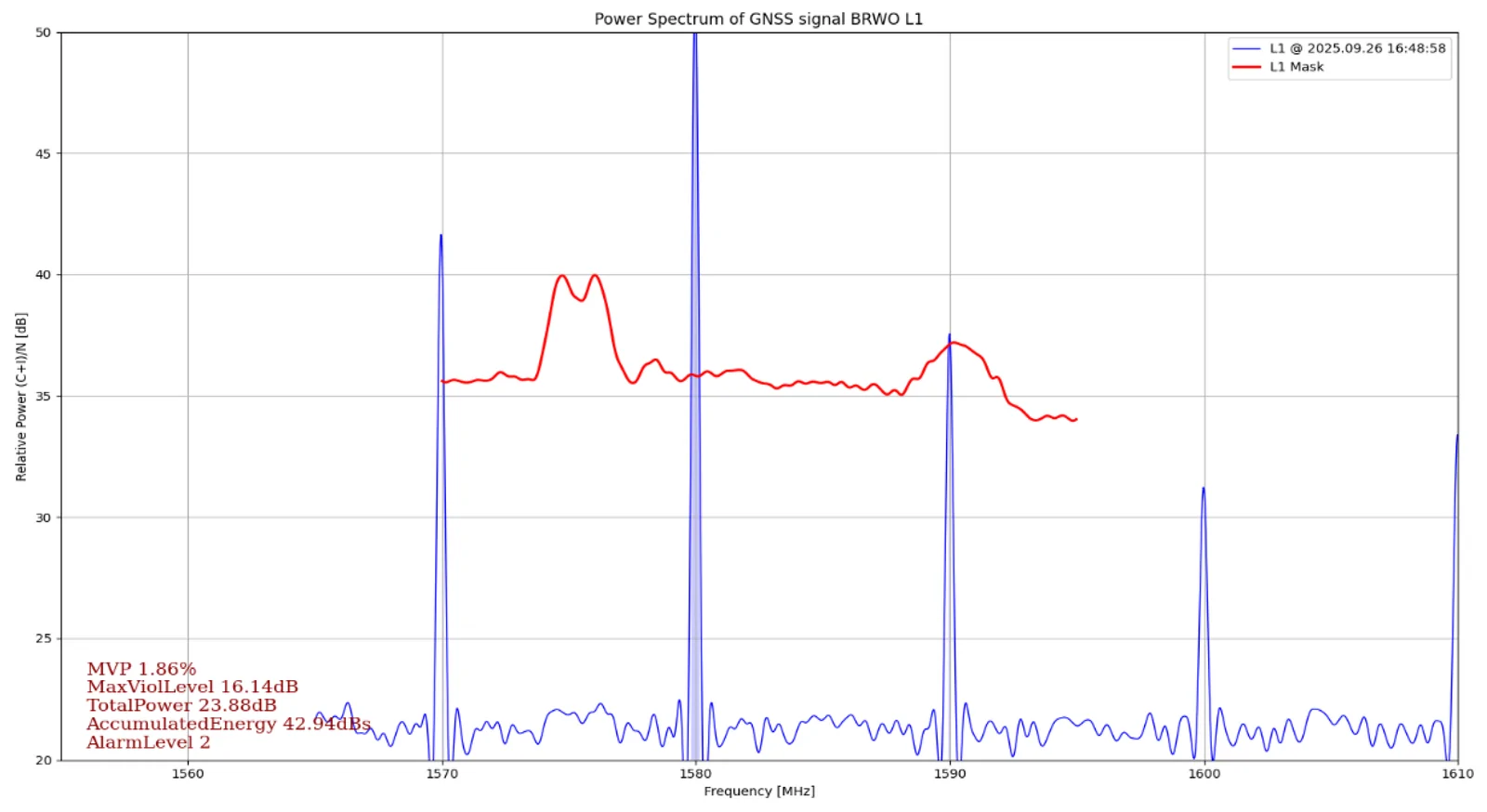
Intermodulation products caused by a Continuous Wave (CW) interference signal at 1580 MHz.

It is equally important to analyze both noise-dominated and signal-dominated parts of the spectrum when identifying disruptions in the frequency bands of GNSS operation. Even if interference affects only noise-dominated sub-bands, it may still disrupt GNSS services due to the formation of intermodulation products. An example of this phenomenon, observed in Braniewo, is presented in Figure 11, where strong interference detected at 1580 MHz caused the Radio Frequency (RF) input block in the GNSS receiver to adjust to the strong input signal which resulted in generation of the intermodulation artifacts at 10 MHz intervals in the spectrum.

Sufficiently strong interference, in addition to generating intermodulation artifacts, exhibits a recurring change in the Automatic Gain Control (AGC) level in the receiver. This effect cannot be neglected, as high-power interference at 1580 MHz can cause the receiver to attenuate the GNSS signal by as much as 15 dB, as demonstrated in Figure 11.

**Figure 12:**
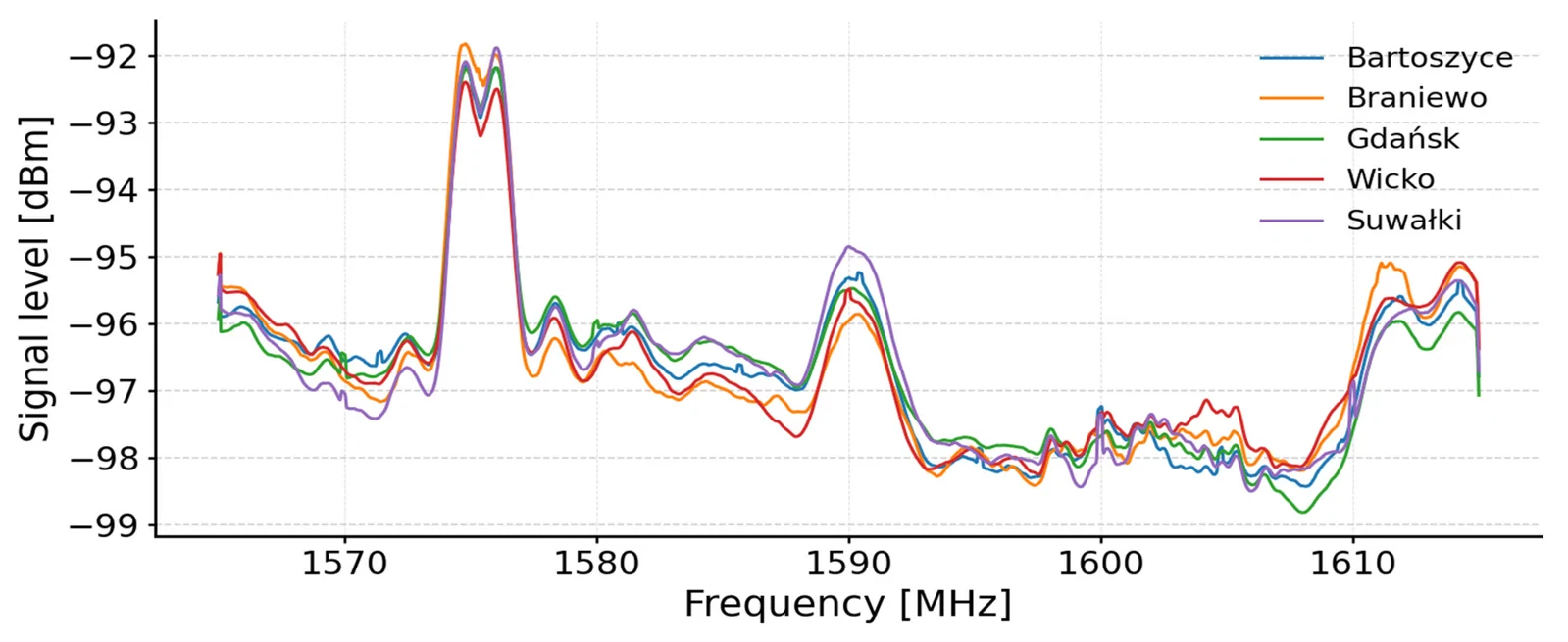
Average spectrum of E1/L1 band at different locations, 16 February 2025.

Spectral traces, collected in interference-free conditions, with fixed AGC level, when analyzed with respect to location exhibited generally similar characteristics across all measurement sites. However, due to differences in antenna paths at different locations, certain variations between spectra were observed, particularly in the noise-dominated portions. Average daily spectra for the E1/L1 frequency band at all five analyzed locations on 16 February 2025 are presented in Figure 12.

**Figure 13:**
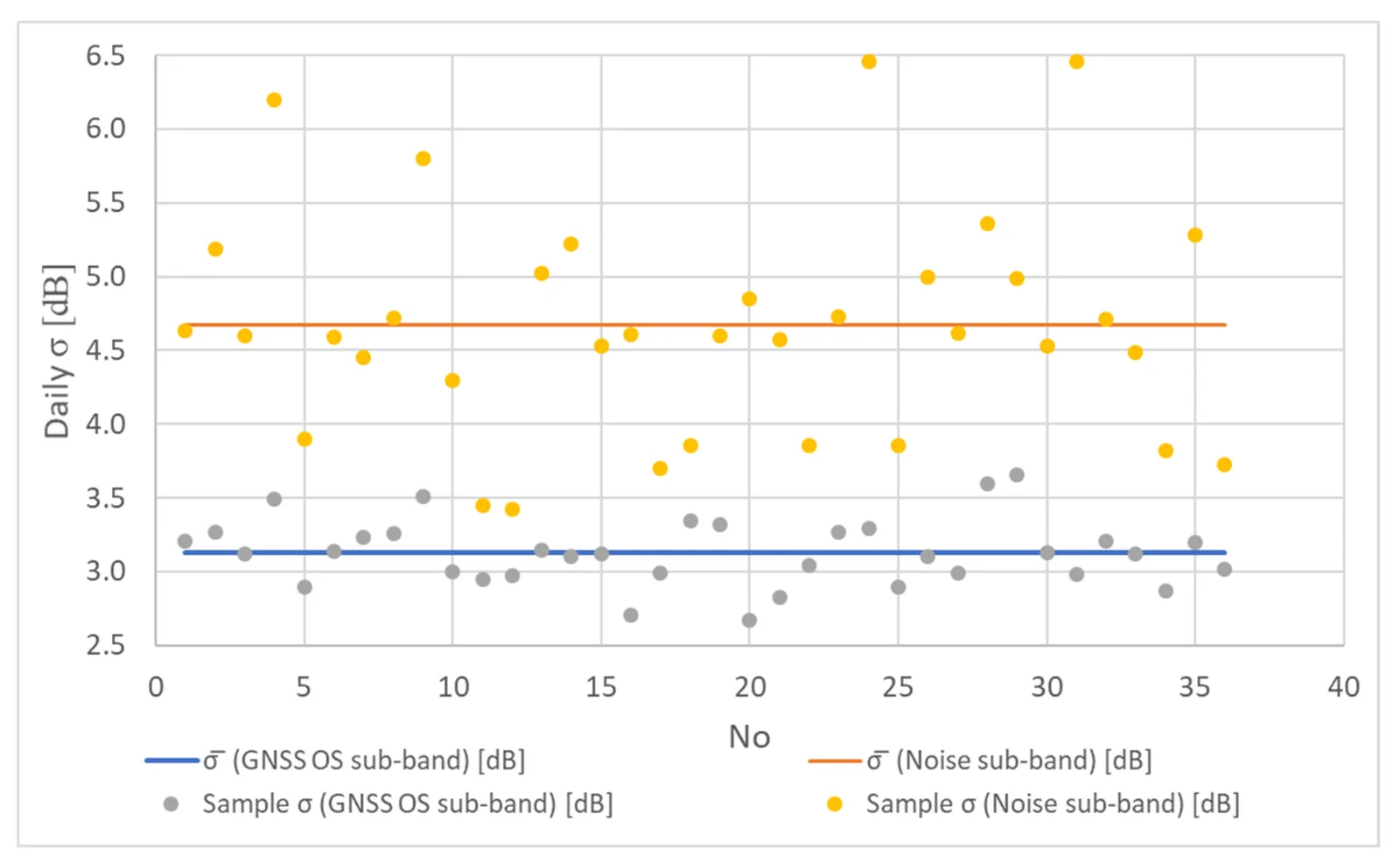
Standard deviation of daily power distribution measurements collected across various monitoring locations.

In order to confirm the accuracy of the simulation methodology, around 100 000 E1/L1 band spectral traces from 4, 6, 7, 11, 12, 13, 14, 18, and 19 March 2025 were collected from stations in Bartoszyce, Gdańsk, Suwałki and Wicko and selected for group comparison of GNSS power variability. Spectral power data was analyzed separately for each of the four locations and for two types of sub-bands: noise-dominated and GNSS OSs. The mean estimated statistical parameters of single spectral traces, based on distributions rescaled as in Equation (28), along with their corresponding standard errors, as well as the mean number of averaged spectral traces for all stations, are presented in Table 4a,b. Additionally, the sample standard deviations of the daily power distributions for No observations are shown in Figure 13.

Based on these results, it is evident that the statistical parameters of GNSS spectral power in the E1/L1 band depend on the analyzed sub-band, which may indicate that deterministic signal components influence the overall received GNSS signal power distribution. If the measured standard deviation values of the GNSS Open Service (OS) sub-band were exclusively considered when calculating the protection margin for the RTGMS according to (17), assuming the aforementioned measured power distribution parameters, a minimum number of averaged spectral traces of N_avg_ = 25, and the corresponding value of parameter k for a 0.1% probability derived from Student’s t-distribution tables, parameter A may vary between 2.1 and 2.4 dB. However, in the case of standard deviation for the noise-dominated sub-band, with identical values for the other parameters, parameter A may vary from 2.7 to 3.6 dB. A comparison between the obtained measurement results and the conducted simulations leads to the conclusion that the measured data exhibit behavior consistent with the simulated data representing the sum of signal distributions with different mean values presented in Chapter 4. Noise-dominated sub-band measurements can also be interpreted as consistent with FFT-based simulations, since they exhibit higher standard deviation as well as negative skewness.

## 6. Conclusions

This study presents methods for characterizing signal power variability within GNSS monitoring systems based on spectral analysis of received signals. It provides approaches for determining statistical parameters of signal power distributions, considering the composite nature of signal components and their respective distributions.

The increasing reliance on GNSS-based services renders systems that merely indicate trends in interference events insufficient, particularly in regions affected by military conflicts. This underscores the importance of systems that enable highly sensitive, long-range interference detection systems that maintain a low false alarm probability. Achieving this requires the introduction of carefully designed protection margins, which can only be properly established through an accurate characterization of the stochastic properties of signals at the receivers.

Based on extensive measurement campaigns and simulations, the authors demonstrate the importance of accurately characterizing the statistical properties of received GNSS signals and highlight the impact of the power distribution parameters of composite signal components on the resulting distribution.

The study focuses on signal components with different statistical distributions, as GNSS monitoring systems operate under diverse conditions and frequently process low-power signals, for which noise power cannot be neglected. This is supported by the introduction in Poland of the nationwide RTGMS system, developed by the NIT in cooperation with GUGiK and funded by the ESA under the NAVISP Element 3 program. This interference detection system is based on a terrestrial network of monitoring stations spread throughout the country.

The analyses in this study are deliberately restricted to the stochastic behavior of signal power components observed under stable Line-of-Sight (LOS) conditions, which can be modeled using a log-normal distribution. The majority of satellite signals variability can be characterized by large-scale fading. This is due to the absence of significant obstacles between satellites and monitoring receivers during signal propagation, making the transmitter–receiver distance the primary factor influencing variability. Similarly, interfering radio signals are predominantly affected by large-scale fading due to the elevated placement of receiver antennas, typically on building rooftops, which results in near-continuous LOS conditions.

The results obtained from both simulations and conducted measurements, specifically, the Monte Carlo simulation results and the comparison of power variability between noise-dominated spectral regions and GNSS OS dominated sub-bands, confirm that differently distributed signal components significantly influence the resulting power distribution at the receiver.

It is noteworthy that while simulations and measurements of summed distributions yield consistent results, discrepancies arise in FFT-based simulations of noise-like signals. It is highly probable that these discrepancies arise from the influence of deterministic signals on the power envelope. This observation, together with differences in statistical parameters associated with variations in component mean power, suggests that the deterministic characteristics of signal components may influence their statistical behavior. Therefore, examination of modulation schemes effect on discussed signals power variability should be the next step for the continuation of this research.

Based on the results obtained in the measurements section, it can be concluded that calculating a protection margin for the RTGMS is more complex than determining a single value if the detection range cannot be compromised and the false alarm probability must be maintained at a very low level.

These analyzes lead to the conclusion that, in order to implement a highly reliable system that enables GNSS interference detection across large areas without triggering a substantial number of false alarms, the traditional approach of using a single protection margin value (as presented, for example, in [34]) for the entire band such as L1 may not be appropriate. Both the statistical parameters and the A values demonstrate that the variability of the power envelope of received signals may differ across certain sub-bands of the analyzed spectrum. Therefore, to maximize detection sensitivity and, consequently, the detection range without increasing the false alarm probability, it may be necessary to calculate varying levels of protection margins across major GNSS frequency bands.

The methods and results presented in this study deliberately focus on the detection of low-power and distant interference. However, the proposed methodology may be excessive for detecting high-power interference in close proximity to RTGMS stations. Such interference clearly causes abnormal adjustments of the receiver’s AGC level, rendering further spectral analysis ineffective. Therefore, the authors suggest that interference detection for high-power signals in close proximity to GNSS receivers at monitoring stations should be based on AGC parameter monitoring.

This paper constitutes an important initial step toward the development of highly accurate spectrum-based long-range interference detection algorithms. Future work may include extending the simulation framework to incorporate deterministic signals that emulate GNSS and potential interference modulations, as well as conducting additional measurement campaigns to analyze signal power spectra in the presence of controlled interference sources.

The RTGMS system described in this paper is also planned for further development. Future work relevant to the subject of this study includes expanding the spectral analysis module. These developments may involve more advanced classification (i.e., additional alarm priority levels), taking into account factors such as the severity of the interference and the services affected. Furthermore, based on the findings of this study, more complex masks are to be implemented, with varying levels across the spectrum calculated and calibrated separately for individual services and noise-dominated segments. Differences in the spectral mask are also intended to incorporate more complex calculations of the safety margin parameter, with multiple levels across the spectrum, accounting for variations in spectral regions dominated by specific signals. It is also planned to implement detection based on AGC monitoring to reduce computational load in the case of strong, non-distant interference.

## Figures and Tables

**Table 1:** Errors in mean values and standard deviations for the LNM methods.

	k-LNM Errors (k = 0.7)		k-LNM Errors (k = 0.5)		t-LNM Errors	
Δσ_in_ [dB]	μ [dB]	σ [dB]	μ [dB]	σ [dB]	μ [dB]	σ [dB]
0	0.2	−0.3	0.3	−0.6	0.0	0.0
1	0.2	−0.2	0.3	−0.5	0.0	0.0
2	0.0	0.2	0.3	−0.2	0.0	0.0
3	−0.5	0.9	0.0	0.4	0.0	0.0
4	−1.2	1.7	−0.6	1.2	0.0	0.0
5	−2.1	2.4	−1.4	2.0	0.0	0.0
6	−2.9	3.1	−2.2	2.7	0.0	0.0

**Table 2:** Skewness and kurtosis values based on Monte Carlo simulations.

Δσ_in_ [dB]	γ	κ
0	0.1	0.0
1	0.2	0.2
2	0.4	0.5
3	0.6	0.8
4	0.8	1.1
5	0.9	1.4
6	1.0	1.6

**Table 3:** Values of skewness and kurtosis for different numbers of averaged spectral traces.

N_avg_	γ	κ
1	−1.15	2.50
5	−0.46	0.36
10	−0.31	0.21
15	−0.28	0.16
20	−0.20	0.07
25	−0.20	0.11
30	−0.18	0.07
35	−0.18	0.08
40	−0.17	0.06
45	−0.15	0.05
50	−0.14	0.07

**Table 4:** Estimated mean parameters of power distributions for E1/L1 sub-bands: (**a**) GNSS OS and (**b**) noise.

**(a)**				
**Station location**	$\bar{N_{avg}}$	$\bar{\sigma }[dB]$	$\bar{\gamma }$	$\bar{\kappa }$
Bartoszyce	50	3.0 ± 0.1	0.0 ± 0.0	0.4 ± 0.4
Gdańsk	105	3.2 ± 0.1	−0.1 ± 0.3	0.5 ± 0.3
Suwałki	113	3.5 ± 0.2	0.0 ± 0.1	1.1 ± 0.5
Wicko	56	3.1 ± 0.1	0.0 ± 0.0	0.2 ± 0.0
**(b)**				
**Station location**	$\bar{N_{avg}}$	$\bar{\sigma }[dB]$	$\bar{\gamma }$	$\bar{\kappa }$
Bartoszyce	50	3.9 ± 0.1	−0.5 ± 0.1	0.6 ± 0.1
Gdańsk	105	4.6 ± 0.1	−0.5 ± 0.1	0.6 ± 0.1
Suwałki	113	5.2 ± 0.1	−0.9 ± 0.3	1.2 ± 0.3
Wicko	56	4.5 ± 0.1	−0.6 ± 0.1	1.6 ± 0.1

## Data Availability

All collected measurement data and simulation results analyzed during the current study are available from the corresponding author on reasonable request.

## Abbreviations

The following abbreviations are used in this manuscript:

GNSS	Global Navigation Satellite System
RTGMS	Real-Time GNSS Monitoring System for Poland
NIT	National Institute of Telecommunications in Poland
GUGiK	Poland’s Head Office of Geodesy and Cartography
ESA	European Space Agency
MSP2G	Mobile GNSS Galileo Measurement Platform
GPS	Global Positioning System
PNT	Positioning, Navigation and Timing
SNR	Signal-to-Noise Ratio
HDOP	Horizontal Dilution of Precision
VDOP	Vertical Dilution of Precision
TDOP	Time Dilution of Precision
I/C	Interference-to-Signal Power Ratio
C/N	Carrier-to-Noise Ratio
RBW	Resolution Bandwidth
RV	Random Variable
CLT	Central Limit Theorem
LNM	Log-Normal Method
FFT	Fast Fourier Transform
OS	GNSS Open Service
RF	Radio Frequency
CW	Continuous Wave
AGC	Automatic Gain Control
LOS	Line-of-Sight
